# The role of naturally acquired intracellular *Pseudomonas aeruginosa* in the development of *Acanthamoeba* keratitis in an animal model

**DOI:** 10.1371/journal.pntd.0011878

**Published:** 2024-01-02

**Authors:** Binod Rayamajhee, Mark Willcox, Fiona L. Henriquez, Ajay Kumar Vijay, Constantinos Petsoglou, Gauri Shankar Shrestha, Hari Kumar Peguda, Nicole Carnt

**Affiliations:** 1 School of Optometry and Vision Science, Faculty of Medicine and Health, UNSW, Sydney, Australia; 2 School of Health and Life Sciences, University of the West of Scotland, Blantyre, Scotland, United Kingdom; 3 Sydney and Sydney Eye Hospital, Southeastern Sydney Local Health District, Sydney, Australia; 4 Save Sight Institute, University of Sydney, Sydney, Australia; Universidade Federal de Sergipe, BRAZIL

## Abstract

**Background:**

*Acanthamoeba* is an environmental host for various microorganisms. *Acanthamoeba* is also becoming an increasingly important pathogen as a cause of keratitis. In *Acanthamoeba* keratitis (AK), coinfections involving pathogenic bacteria have been reported, potentially attributed to the carriage of microbes by *Acanthamoeba*. This study assessed the presence of intracellular bacteria in *Acanthamoeba* species recovered from domestic tap water and corneas of two different AK patients and examined the impact of naturally occurring intracellular bacteria within *Acanthamoeba* on the severity of corneal infections in rats.

**Methodology/Principal findings:**

Household water and corneal swabs were collected from AK patients. *Acanthamoeba* strains and genotypes were confirmed by sequencing. *Acanthamoeba* isolates were assessed for the presence of intracellular bacteria using sequencing, fluorescence *in situ* hybridization (FISH), and electron microscopy. The viability of the bacteria in *Acanthamoeba* was assessed by labelling with alkyne–functionalized D–alanine (alkDala). Primary human macrophages were used to compare the intracellular survival and replication of the endosymbiotic *Pseudomonas aeruginosa* and a wild type strain. Eyes of rats were challenged intrastromally with *Acanthamoeba* containing or devoid of *P*. *aeruginosa* and evaluated for the clinical response. Domestic water and corneal swabs were positive for *Acanthamoeba*. Both strains belonged to genotype T4F. One of the *Acanthamoeba* isolates harboured *P*. *aeruginosa* which was seen throughout the *Acanthamoeba*’s cytoplasm. It was metabolically active and could be seen undergoing binary fission. This motile strain was able to replicate in macrophage to a greater degree than strain PAO1 (*p*<0.05). Inoculation of *Acanthamoeba* containing the intracellular *P*. *aeruginosa* in rats eyes resulted in a severe keratitis with increased neutrophil response. *Acanthamoeba* alone induced milder keratitis.

**Conclusions/Significance:**

Our findings indicate the presence of live intracellular bacteria in *Acanthamoeba* can increase the severity of acute keratitis *in vivo*. As *P*. *aeruginosa* is a common cause of keratitis, this may indicate the potential for these intracellular bacteria in *Acanthamoeba* to lead to severe polymicrobial keratitis.

## Author summary

In recent years, *Acanthamoeba* spp. have become an increasingly important human pathogen, causing serious, debilitating, and sometimes deadly infections. It can cause a rare but severe and potentially blinding corneal infection known as *Acanthamoeba* keratitis (AK), which is extremely painful, difficult to diagnose, and treat. In a remarkable dual role, *Acanthamoeba* species act as phagocytic predator, consuming other microbes, but also as environmental hosts for a wide range of bacteria. With the increasing prevalence of coinfections among AK patients, which often lead to the severe outcomes, it is important to identify the intracellular bacteria hosted by *Acanthamoeba* spp. Using a series of experiments we confirmed the presence of intracellular *P*. *aeruginosa* in an *Acanthamoeba* strain isolated from AK patient domestic tap water. Our findings showed that amoeba adapted *P*. *aeruginosa* exhibited enhanced intracellular survival and replication in human monocyte derived macrophages (hMDMs) compared to the wild–type strain PAO1. Here, for the first time, we investigated the role of naturally acquired viable intracellular *P*. *aeruginosa* in the development of *Acanthamoeba* keratitis in rats. The presence of intracellular bacteria in *Acanthamoeba* resulted into acute keratitis, highlighting the importance of identifying endosymbionts harbored by *Acanthamoeba* for accurate differential diagnostics and prognostic evaluations of *Acanthamoeba* keratitis.

## 1. Introduction

*Acanthamoeba*, a widely distributed heterotrophic protist, is described a predator, a reservoir, and a host in various environments for numerous bacteria, fungi, and giant viruses [1, 2]. The feeding stage of *Acanthamoeba*, trophozoites, actively consume various microbes and small organic particles [3]. *Acanthamoeba* species have the ability to inhabit various water systems, including household tap water, where they graze on microbial biofilms to acquire nutrition, protection, and facilitate their dissemination [4–7].

If transferred to the eye, *Acanthamoeba* can cause a progressive keratitis, accounting for approximately 0.5% to 10% of global microbial keratitis cases [8–11]. Contact lens users in developed countries comprise over 85% of reported cases of *Acanthamoeba* keratitis (AK) [12–15]. Water contamination is the primary risk factor for AK associated with contact lens use [4]. In countries where contact lens wear is not common, eye injuries resulting from exposure to wet soil or contaminated water serve as significant sources of AK infection [13,16]. The global annual incidence of AK is 2.9 cases per million people, with India having the highest rate at 15.2 cases per million population [8]. *Acanthamoeba* infection in the cornea can persist for extended periods, and in some patients, removal of eye contents (evisceration) was considered as last resort for disease resolution due to its severity [12,17].

There have been a few reported cases of keratitis coinfections involving *Acanthamoeba* with fungi or bacteria [14,18–22]. In a retrospective study of 110 cases of AK, 23.6% of specimens showed positive bacterial growth, 7.3% exhibited fungal growth, and 9.3% of patients tested positive for herpes simplex virus (HSV) in PCR tests [14]. Similarly, bacterial coinfection was observed among 63.3% of AK patients followed by fungal (10.3%), and HSV (9.4%) treated in a tertiary medical centre in Austria [21]. Bacterial culture has identified a wide range of bacteria associated with coinfections including *Bacillus cereus*, *Staphylococcus haemolyticus*, *S*. *epidermidis*, *Propionibacterium acnes*, *Enterobacter cloacae*, *B*. *simplex*, and *S*. *aureus* [21]. Saad et al. (2018) noted coinfections in 89% of cases with 17 involving both bacteria and fungi among the 102 culture confirmed AK cases in Egypt [19]. Surprisingly, high proportions of isolated bacteria and fungi were able to produce biofilms [19]. According to a recent review conducted in South India, coinfections were found in over 50% of AK cases, with fungal coinfection observed in 34% of cases and bacterial coinfection in 14% of cases [18].

A number of studies indicate a potential enhancement of *Acanthamoeba* virulence in presence of bacterial endosymbionts [20,23,24]. The presence of intracellular bacteria in corneal isolates of *Acanthamoeba* spp. has been linked to lower visual acuity, longer symptom duration, delayed or misdiagnosis, epithelial defect, hypopyon, and stromal infiltrates [20,25]. Additionally, in a rabbit model of AK, the severity of infection was increased following acquisition of *P*. *aeruginosa* by *A*. *castellanii* (ATCC 50492) [26]. *Acanthamoeba* with endosymbiotic gram–negative bacteria or *Chlamydia*–like bacteria resulted in quicker production of cytopathic effects (CPEs) on a fibroblast monolayer [23].

It is important to assess the role of naturally acquired intracellular bacteria in the development of AK *in vivo*. To our knowledge, no study has investigated the severity of AK infection in rat corneas using *Acanthamoeba* with a naturally acquired bacterial population residing within. More broadly, there are limited studies that examine the viability of intracellular bacteria in amoebal hosts using both molecular and culture–based assays concurrently. Therefore, this study aimed to identify viable bacterial endosymbionts in *Acanthamoeba* strains obtained from corneal swabs and domestic tap water of AK patients and examine whether the presence of intracellular bacteria in *Acanthamoeba* trophozoites affects the severity of corneal infection using experimental AK rat model.

## 2. Materials and methods

### Ethics statement

This study protocol was reviewed and approved by the Human Research Ethics Committee (HREC), Southeast Sydney Local Health District (SESLHD), Australia (2020/ETH02726); Animal Care and Ethics Committee, UNSW, Sydney, Australia (22/67A), and the UNSW Human Research Ethics Committee, Sydney, Australia (ETH00520, for blood collection from healthy donors).

### Axenic cultivation of *Acanthamoeba* isolates

*Acanthamoeba* isolates were obtained by culturing swabs from the cornea of one AK patient and a water sample from the domestic tap of another AK patient. These patients were treated at the Sydney Eye Hospital, Australia (ethics number: 2020/ETH02726). *Acanthamoeba* isolates obtained through primary isolation were cultured axenically in 12–well culture plates at 32°C in peptone–yeast–glucose (PYG) broth using previously described methods with some modifications [20,27,28]. Briefly, corneal swab collected form an AK patient along with quarter–strength Ringer’s solution in a 1.5 ml sterile Eppendorf tube was directly inoculated onto non-nutrient agar (NNA; mM NaCl, 1 mM KH_2_PO_4_, 40 mM CaCl_2_, 0.5 mM Na_2_HPO_4_, and 20 mM MgSO_4_) plate preseeded with heat killed *Escherichia coli* (ATCC 10798). The plate was incubated for up to four weeks at 32°C and regularly observed for the appearance of *Acanthamoeba* trophozoites or cysts using an inverted light microscope (IX73 Inverted Microscope, Olympus). Following initial isolation, the *Acanthamoeba* strain was cultured on fresh NNA without *E*. *coli*, then transferred into PYG broth [proteose peptone (20 gm), yeast extract (20 gm), glucose (18 gm), NaCl (120 mg), MgCl_2_-6H_2_0 (3 mg), Na_2_HPO_4_ (142 mg), KH_2_PO_4_ (136 mg), CaC1_2_ (3 mg), and FeSo_4_ (3 mg) in 1000 ml milli–Q water, pH 6.5]. The same procedure was followed to recover *Acanthamoeba* from domestic tap water sample, with the exception that 15 ml of water in the test tube was vortexed at 1000g for 10 mins, and subsequently, the pellet was inoculated onto the NNA plate. To prevent culture contamination and eliminate extracellular bacteria, each strain was cultured in 4 mL of PYG medium supplemented with 250 μl/mL penicillin–streptomycin (Thermo Fisher, USA), i.e., axenic growth conditions. The antibiotics used to maintain the axenic culture either do not penetrate eukaryotic cells (penicillin) or are concentrated but inactivated in the low pH environment of phagolysosomes (streptomycin) [29]. Therefore, these act exclusively on extracellular bacteria if present in the PYG, preserving the viability of intracellular bacteria. However, to assess the presence of contaminant bacteria in the penicillin–streptomycin containing PYG medium, 15 μl aliquot of PYG was inoculated onto trypticase soy agar (Becton, Dickinson, and Company, Sparks, MD, USA) and incubated for 48 hours at 37°C. After incubation, the growth of any bacteria on the agar plates was excluded from the study. In addition, the culture medium was refreshed with freshly prepared PYG every 24 hrs until the trophozoites were harvested. Amoebal DNA was extracted using Chelex resin (MB Chelex–100 resin; Bio–Rad Laboratories, CA, USA) as previously described [30] and PCR was used to amplify the highly variable DF3 region of 18S rRNA (*Rns* gene) using primers (JDP1 and 2) and cycle conditions that had been previously established [25]. The amplified products were Sanger sequenced and the sequences were aligned using MUSCLE algorithm to construct a phylogenetic tree with MEGA11 [31].

### A. Characterization of naturally acquired intracellular bacteria by *Acanthamoeba*

#### Detection of intracellular bacteria

Axenically grown *Acanthamoeba* isolates were screened for the presence of intracellular bacteria in trophozoites. Trophozoites were harvested and passed at least 10 times through 29G ultrafine syringe (BD, Sparks, MD, USA) to completely lyse them. The lysate was centrifuged at 500g for 5 mins to acquire the cell pellet. Total genomic DNA (gDNA) was extracted using DNeasy blood and tissue kit following manufacturer’s instruction (Qiagen, GmbH, Hilden, Germany). The presence of intracellular bacteria in both *Acanthamoeba* isolates was first assessed using eubacteria 16S rRNA PCR primers (341Fw and 785Rv) to amplify V3–4 as previously described [32]. The gDNA positive for 16S rRNA PCR was sent for Sanger sequencing to identify the genus of intracellular bacteria. Furthermore, 16S rRNA–positive amoebal cells were lysed using 500μl of 1% Triton–X100 for 1 min (Sigma–Aldrich, St. Louis, USA), after which the mixture was pelleted and washed twice with 1 mL of 1X phosphate–buffered saline (PBS; Sigma–Aldrich, USA). The cell pellet was cultured on trypticase soy agar (TSA; Becton, Dickinson, and Company, Sparks, MD, USA) to determine the ability of intracellular bacteria to grow outside of the amoebal host. The pure colonies of bacteria were used for MALDI–TOF mass spectrometry (Bruker MALDI Biotyper, Bremen, Germany) to confirm the bacterial species.

#### Fluorescent *in situ* hybridization (FISH) assay

A previously described protocol was used for fluorescent *in situ* hybridization [1,20]. Double FISH was performed with a probe that specifically binds to the complementary sequence of 16S rRNA of the targeted intracellular bacteria, in conjunction with a probe that hybridized to the 16S rRNA of the majority of bacteria (5’–GCTGCCTCCCGTAGGAGT–3’, EUB338, probeBase, Wien, Austria). A fluorescein isothiocyanate (FITC) dye–conjugated oligonucleotide probe (5’–GGTAACCGTCCCCCTTGC–3’, pB–383) was used that specifically binds to *P*. *aeruginosa* strain AK1–PA, identified in *Acanthamoeba* AK1. Cy5 labelled EUK516 probe was used to hybridize with amoebal 18S rRNA (S1 Table). The hybridization step was performed in the dark at 46°C for 90 mins. FISH–stained slides were observed using confocal microscope (Olympus FV1200) and images were subsequently analysed in ImageJ.

#### Transmission electron microscopy (TEM)

Axenically cultured trophozoites were harvested from culture medium, washed three times with 1X PBS (2.7 mM KCl, 1.4 mM NaCl, 10 mM Na_2_HPO_4_ and 1.8 mM KH_2_PO_4_, pH 7.0) and pelleted by centrifugation (600g, 5 min). The cell pellets were fixed in 2.5% ice–cold glutaraldehyde in 0.2 M sodium phosphate buffer at 4°C overnight. After rinsing with 0.1 M sodium phosphate buffer, the samples were post–fixed in 1% osmium tetroxide with 1.5% potassium ferrocyanide in 0.2 M sodium buffer using a BioWave Pro+ Microwave Tissue Processor. Dehydration was carried out using graded ethanol series (30%, 50%, 70%, 80%, 90%, and 100%) followed by resin infiltration (Procure, 812). The resin–embedded samples were polymerized at 60°C for 48 hrs. Ultrathin sections of 70 nm were cut using a diamond knife (Diatome, Nidau, Switzerland) and placed on carbon–coated copper slot TEM grids. The grids were post–stained with 2% uranyl acetate and lead citrate. Two grids were prepared for each sample and imaged using an ultra–high–resolution scanning JEOL TEM–1400 operating at 100 kV.

#### Motility, *exoU* and *exoS* gene detection in *P*. *aeruginosa* AK1–PA

Swimming, swarming, and twitching motilities were evaluated using motility agar supplemented with 0.3%, 0.5%, or 1% (wt/vol) agarose (Bacto; BD Biosciences, USA) as described previously [33]. In 6–well plates, five ml of motility agar was dispensed into the wells and allowed to dry. For the evaluation of twitching motility, *P*. *aeruginosa* AK1–PA was inoculated at the base of the plate using 10 mL pipette tips, while for the assessment of swimming and swarming, it was inoculated in the middle of the agar. Incubation periods of 48 hrs at 25°C were maintained for twitching and swarming plates, whereas swimming plates were incubated for 24 hrs prior to imaging. The wild–type strain *P*. *aeruginosa* PA01 (ATCC 15692) was used as a prototroph for comparing the motility of the AK1–PA. The diameters of the migration zone were analysed semi–quantitatively using ImageJ. The motility was performed in triplicate (*n* = 3).

The presence of type III secretion system (T3SS) effectors genes such as *exoU* and *exoS* in AK1–PA strain was assessed using primers and PCR conditions (S2 Table), as described elsewhere [34,35].

#### AlkDala labelling of *P*. *aeruginosa* in amoebal host (AK1)

**Fig 1:**
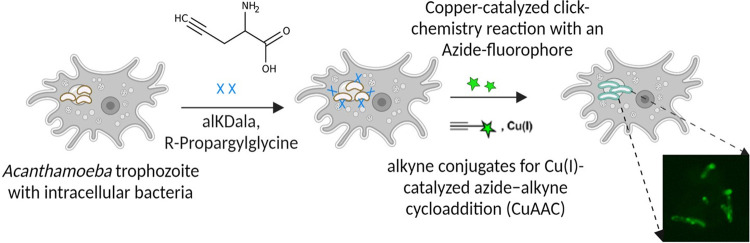
Graphic presentation of alkDala (represented by ‘x’) labelling of intracellular bacteria in amoebal host [36]. Trophozoites containing intracellular bacteria were exposed to AlkDala, facilitating its incorporation into the cell wall peptidoglycan, followed by a copper–catalysed click–chemistry reaction with an Azide–fluorophore (represented by green stars) to identify bacteria carrying the incorporated probe.

Labelling of live bacteria using alkDala (alkyne functionalized D–alanine), a biorthogonal probe, was adapted to access the viability of *P*. *aeruginosa* inside amoebal host [36,37]. Axenically maintained trophozoites were incubated in a solution containing 10 mM alkDala (Thermo Scientific, Altrincham, UK) at 30°C with shaking for 3 hrs. After alkDala incubation, the cell pellet was harvested and transferred to poly–L–lysine coated slides (Thermo Scientific, Braunschweig, Germany) for 30 mins at 25°C. The slides with adhered trophozoites were then fixed in pre–chilled 70% EtOH for 20 mins at –20°C. The slides were washed with 1X PBS, and the amoebal cells were permeabilised in PBS with 0.5% Triton–X100 for 15 mins at 25°C, followed by washes in PBS with 0.1% Triton–X100 and 3% BSA (3 times, 5 mins each) at 25°C with shaking. Permeabilised trophozoites were transferred to the click–labelling cocktail reaction (S3 Table) with AFDye 488 Azide for 30 mins in dark with shaking at 25°C according to manufacturer’s instructions (Click Chemistry Tools, Scottsdale, AZ, USA). The reaction cocktail was washed using wash buffer provided with kit and PBS was used for final wash. AlkDala–labelled trophozoites were observed under a confocal microscope (Olympus FV1200) (**Fig 1**).

#### Intracellular survival of *P*. *aeruginosa* AK1–PA in human macrophages

Human monocyte–derived macrophages (hMDMs) were harvested from an immunocompetent male healthy donor and cultured in RPMI 1640 (Gibco Life Technologies) containing 2mM glutamine and 100 μg/mL penicillin–streptomycin, following previously described protocols [38,39]. Briefly, total white blood cells (WBCs) were collected from healthy donor whole blood using a Ficoll–Paque plus gradient (Amersham Biosciences AB, Uppsala, Sweden). The isolated WBCs were then suspended in RPMI with 10% heat inactivated human serum (HIHS) and incubated in 12–well low adherence plates for four days. Washing was performed with warm PBS to remove lymphocytes and neutrophils and RPMI with 10% HIHS was refreshed every second day. Adherent cells were harvested and plated into appropriate wells for the experiment in RPMI with 5% HIHS for 1 day. The medium was changed to RPMI containing 1% HIHS for one day then hMDMs were ready for experiments.

To study the uptake and intracellular survival dynamics of *P*. *aeruginosa* strain AK1–PA and wild–type PA01 within primary macrophages, overnight cultures of both isolates were introduced to hMDMs (8×10^4^ cells/well) in a 24–well plate containing RPMI with 5% HIHS. The infection was conducted at a multiplicity of infection (MoI) of 10:1, and the culture condition was maintained at 37°C with 5% CO_2_. After 1 hr of coincubation, the medium was replaced with RPMI containing 300μg/mL gentamicin to kill unengulfed and remaining extracellular bacteria and incubated for 2 hrs as described previously [38,39]. After thorough washing to remove gentamicin, the infection progressed for up to 24 hrs. The host cells (hMDMs) were lysed with 0.02% Triton X–100 (v/v) at 3, 6, 12, and 24 hrs post–infection (p.i.) and the *P*. *aeruginosa* CFUs counts were determined by plating serial dilutions onto TSA (trypticase soy agar; Becton, Dickinson, and Company, Sparks, MD, USA) to quantify surviving intracellular bacteria. The hMDMs monolayers were permeabilized and fixed in 100% methanol at –20°C for 5 mins at 3 and 12 hrs p.i. Subsequently, *P*. *aeruginosa* specific probe (pB–383) was used for hybridization. To stain the nuclei, Prolong Diamond Antifade with DAPI (Thermo Fisher Scientific) mounting medium was applied. Confocal microscopy was used to examine the monolayers, and each experiment was conducted in triplicate. At 3, 6, and 12 hrs p.i., the monolayers were permeabilized and fixed at –20°C in 100% methanol for 5 mins followed by hybridization using *P*. *aeruginosa* specific probe (pB–383) then Prolong Diamond Antifade with DAPI (Thermo Fisher Scientific) mounting medium was used to stain the nuclei. Monolayers were examined by confocal microscopy and each experiment was performed in triplicate. The numbers of intracellular bacteria in macrophage cells were enumerated, and the intracellular doubling time of AK1–PA and PA01 were compared.

### B. Experimental keratitis in rats

#### Animals and inoculation procedure

Prior to the start of the experiments, ethics approval was granted from the Animal Care and Ethics Committee of the University of New South Wales (UNSW), Sydney, Australia. All procedures were performed according to the Animal Care and Ethics Committee guidelines (ACEC– 22/67A). The animal experiment consisted of two phases. In the first phase, original *Acanthamoeba* strains AK1 and AK11 were used, while in the second phase *Acanthamoeba* (labelled as AK10) and *P*. *aeruginosa* (AK1–PA) re–isolated from corneal homogenate of the first phase rats were used. In this study, a total of 14 Wistar rats aged 8 to 10 weeks and weighing 283 to 300 grams (SD ± 5.1) were used.

**Fig 2:**
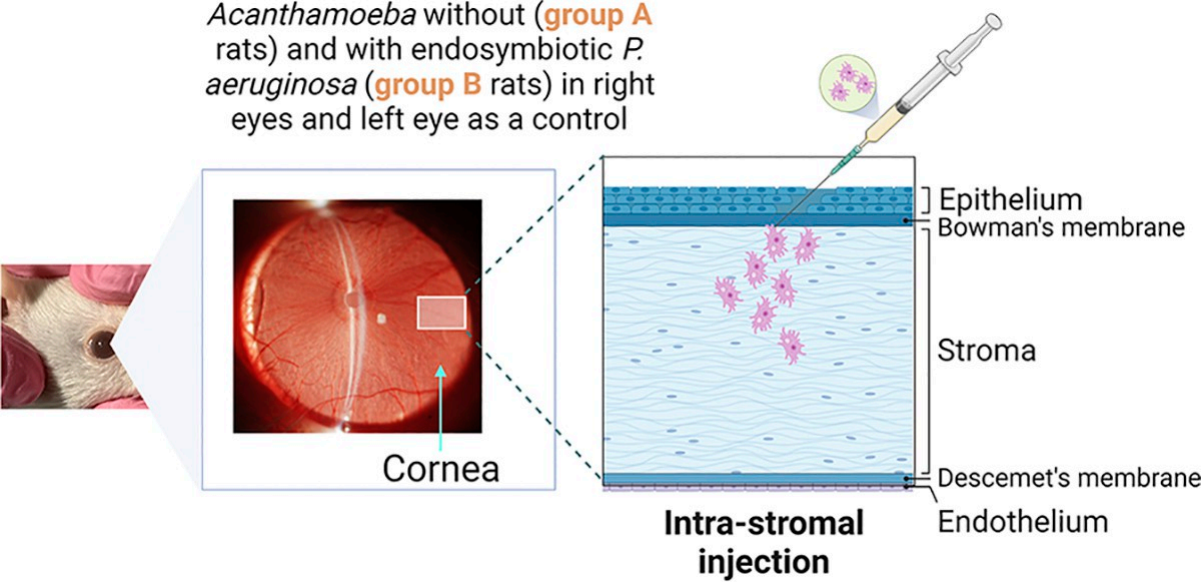
Trans–corneal inoculation of *Acanthamoeba* suspension: Diagrammatic illustration of intrastromal injection in rat’s cornea using 10μL Hamilton surgical micro–syringe with 33G needle (first phase of the experiment). The intrastromal injection was precisely performed in four steps [40]; (**i**) A surgical forceps was used for proptosis of the eye by carefully retracting the lower and upper lids, (**ii**) Creation of a small intrastromal pocket with 33G needle in the mid–peripheral cornea, (**iii**) Inserting a smaller portion of needle and advancing it intrastromally toward the corneal centre, and (**iv**) Gentle inoculation of amoebal suspension (~2μl).

After a week of acclimation in the animal facility, all rats were screened by slit lamp with a digital camera (Nikon, D100) for any pre–existing corneal injuries. Rats with normal corneas were sedated by administering a combination of ketamine (100mg/kg) and xylazine (10mg/kg) via intraperitoneal injection using a 25G needle (Terumo Corporation, Tokyo, Japan). A 10 μL Hamilton surgical syringe with 33G needle was used to inject precisely 2 μL of amoebal suspension (10^4^ trophozoites) keeping the needle at approximately 30° angle into the right eye’s corneal stroma using surgical binocular loupes (**Fig 2**). The left eye of each rat received a mock inoculation of PBS (2 μL) as a control. During recovery from anaesthesia, rats were placed on heated recovery pads in a dark room and their breathing rate was examined regularly until recovery. In addition, normal saline (Pfizer, Australia) was used to prevent the eyes of rats from drying out during anaesthesia.

In the first phase of experiment, rats were divided into two groups. Group A (*n* = 4) received *Acanthamoeba* T4 strain AK11 devoid of any intracellular bacteria, while group B (*n* = 4) received *Acanthamoeba* T4 strain AK1 that had been confirmed to contain viable intracellular *P*. *aeruginosa*. *Acanthamoeba* and *P*. *aeruginosa* were re–isolated from the corneal homogenates of group B rats. However, it was interesting to observe no intracellular bacteria in the trophozoites of AK1 strain, as indicated by FISH staining and that was relabelled as strain AK10. In the second phase of experiment, *Acanthamoeba* (AK10) and *P*. *aeruginosa* AK1–PA re–isolated from corneal homogenate of group B rats from the first phase were inoculated into rats of group C (*n* = 3) and D (*n* = 3), respectively.

#### Clinical evaluation, end point, and pathological analysis of AK

**Fig 3:**
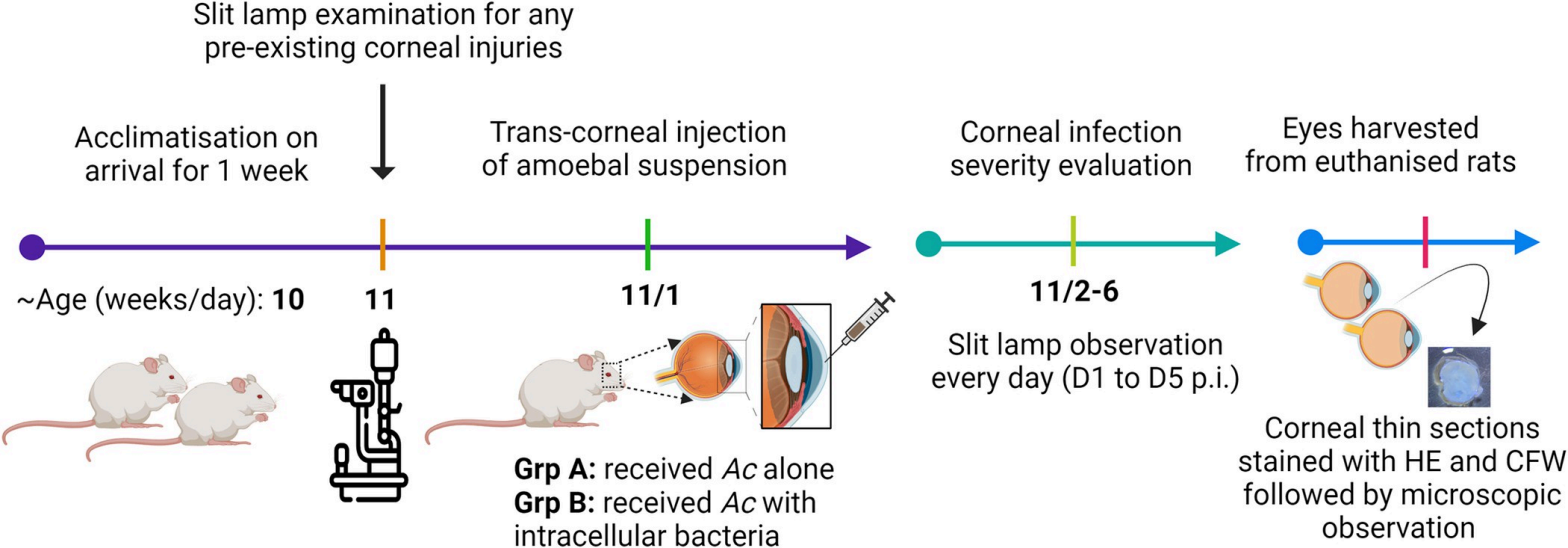
Experimental flow chart of AK rat model. After a week of acclimatisation, intrastromal inoculation of *Acanthamoeba* (*Ac*) suspension (~2μl, ~10^4^ trophozoites) was performed precisely using a 10μL micro–syringe with 33G needle. Slit lamp observation was performed from day 1 to 5 to examine the clinical features of keratitis progression. After infection period, eyes were harvested for pathological analysis of AK.

On days 1, 2, 3, 4, and 5 post–infection (p.i.), the anterior segment of both eyes in all rats was examined using a slit lamp with and without fluorescein (BioGlo fluorescein strips, HUB Pharmaceuticals, AZ, USA). Each cornea was assessed and assigned a grade ranging from 0 to 4, based on the density and area of opacity, conjunctivitis, surface regularity, and discharge (S4 Table) as described previously [41]. The clinical endpoint was defined as either weight loss of ≥20% compared to initial weight and/or the presence of severe keratitis, if the cornea remained clear or showed mild infection during the study period on the sixth day p.i. [42]. Upon reaching the clinical endpoint, the rats were euthanised by using CO_2_ gas exposure with a gradual–fill procedure and both eyes were harvested for downstream analysis (**Fig 3**). Both eyes of one rat from each group were used to prepare corneal homogenates using a sterile homogeniser; the right eye for culturing and recovering the inoculated strain and left eye to screen any growth from uninfected cornea.

Infected and uninfected corneas were dissected from right and left eyes under a dissection microscope (NSZ–405 Zoom Stereo Microscope with camera and illuminator) and stored at 4°C using 30% sucrose in 0.1M PBS (pH 7.4) until sectioning. Paraffin sections were made on a cryostat (CryoStar NX70, Thermo Scientific) for hematoxylin–eosin (H&E, 5μm) and calcofluor white (CFW, 10μm) staining to observe the pathological process of AK in rat corneas and the presence of *Acanthamoeba* cysts in corneal sections, respectively. Each cornea was examined using a minimum of 10 sections. To confirm *Acanthamoeba* cyst–like structures stained with CFW, suspected cells were collected using MicroBeam Laser Microdissection (Zeiss) followed by PCR of the collected cells using *Acanthamoeba* specific primer (JDPFw/Rv).

#### Myeloperoxidase assay

Myeloperoxidase (MPO) activity, which corresponds to the quantity of polymorphonuclear neutrophils (PMNs) present, was assessed as described elsewhere [43]. Briefly, 0.5% (w/v) cetyltrimethylammonium bromide (CTAB) was added to a 90 μl aliquot of corneal homogenate prepared as described above. Triplicate samples of corneal homogenate from each rat group were sonicated and subjected to three freeze–thaw cycles before centrifuged at 8000×g for 20 mins in a cold (4°C) microfuge. Supernatants of 10 μl were pipetted into a 96–well plate, followed by addition of PBS (90 μl/well) containing 0.002% H_2_O_2_ and 0.0167g/100ml of o–dianisidine dihydrochloride for the reaction. The absorbance change at 3 mins was measured at 460 nm and compared to a standard curve. The standard curve was generated using purified myeloperoxidase (Planta Natural Products, Vienna, Austria). The absorbance readings were expressed as relative units of MPO activity (PMNs/cornea).

## 3. Results

### 3.1 Genotyping and phylogenetic position of *Acanthamoeba* strains

Sequences of the *Rns* genes from the two *Acanthamoeba* strains AK1 and AK11 used this study were aligned using the MUSCLE algorithm and compared to the NCBI reference strains to confirm genotypes. GenBank accession numbers of AK1 and AK11 are OR263302 and OR263297, respectively. A neighbour–joining phylogenetic tree with 1,000 bootstraps was constructed using Kimura parameter and reference sequences from genotypes T1, T2, T3, T4 (A–G), T5, T6, T12, T13, and T23. Both isolates assessed in this study belong to genotype T4F, indicating they are very closely related allelic forms of the *Rns* with shared features and minor distinctions between them (S1 and S3 Figs).

### 3.2 Molecular detection and identification of intracellular bacteria in *Acanthamoeba*

The 16S rRNA PCR showed the presence of intracellular bacteria in the *Acanthamoeba* strain AK1 which had been isolated from the domestic tap water of an AK patient (S1 and S2 Figs). Amplicon sequencing was performed to identify the bacteria, and the blast_n_ analysis confirmed the bacteria as *P*. *aeruginosa* (GenBank accession number OR297627). Additionally, MALDI–TOF MS analysis confirmed the strain as *P*. *aeruginosa* (score = 1.99; S5 Table). Phylogenetically, the *P*. *aeruginosa* AK1–PA was closely related to previous isolates of *P*. *aeruginosa*, mostly obtained from environmental samples such as guano, soil, water, cloaca of *Bothrops insularis*, and intestinal tract of termites (S4 Fig).

### 3.3. Detection and intracellular localization of intracellular bacteria in *Acanthamoeba* by FISH and TEM

**Fig 4:**
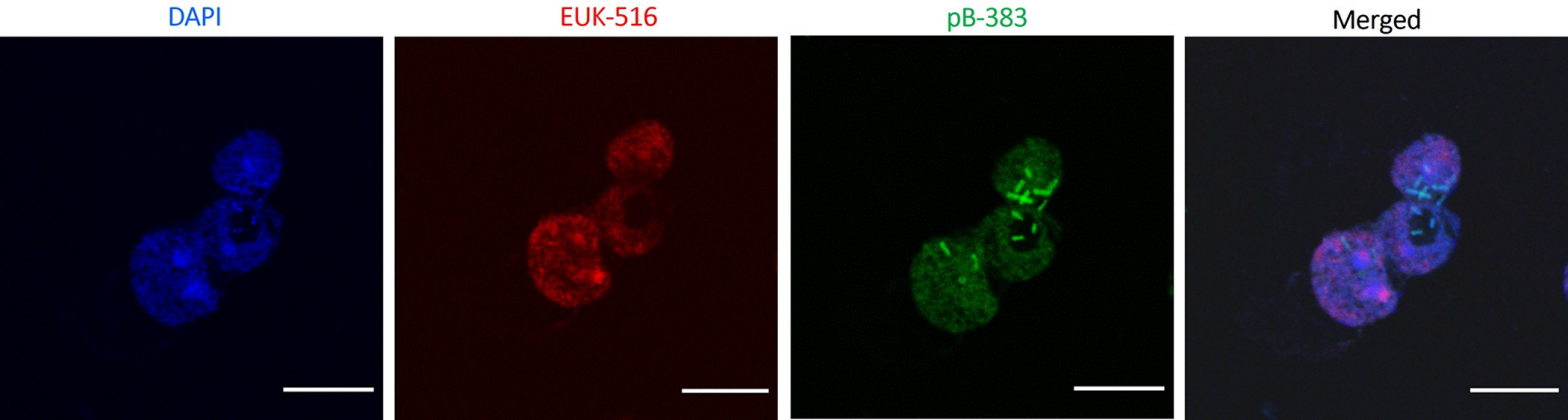
Representative confocal images of hybridization assay showing the presence of *P*. *aeruginosa* in *Acanthamoeba* trophozoites (AK1). Probes EUK516 conjugated with Cy5 (red), targeting *Acanthamoeba*, and pB–383 conjugated with FITC (shown in green) targeting *P*. *aeruginosa* 16S rRNA were used in double FISH assay. DAPI was used in mounting medium when visualized by a fluorescence microscope. White arrow indicates rod–shaped bacterial cells. Scale bar in each image represents 10 μm.

Positive hybridization was observed with a fluorescent dye (FITC)–conjugated DNA probe specific to *P*. *aeruginosa* in *Acanthamoeba* AK1 isolated from domestic tap water of an AK patient (**Fig 4**). The bacterial cells were present throughout the cytoplasm of the trophozoites and were observed in all amoebal cells in the population. Confocal Z–stack images also confirmed the presence of *P*. *aeruginosa* cells inside *Acanthamoeba* trophozoites instead of sitting on the host surface (S5 Fig). This observation showed an average of 4±2.2 bacteria (mean±SD) per trophozoite.

**Fig 5:**
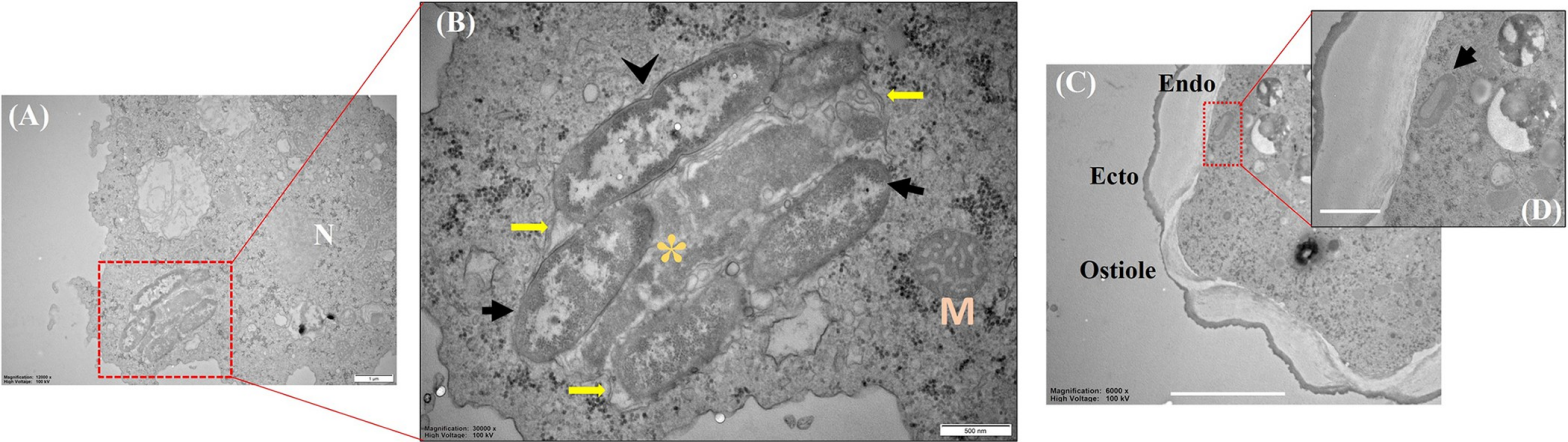
Representative transmission electron micrographs of *Acanthamoeba* (AK1) and its intracellular bacteria (AK1–PA). (A) Overview of a trophozoite with intracellular *P*. *aeruginosa*. (B) Higher magnification showing the rod–shaped intracellular bacteria enclosed within phagolysosome like structure. A bacterial cell is in the process of binary fission. (C–D) Rod shaped bacteria like structure was seen inside empty cyst. Symbols: M: Mitochondria; N: Nucleus; Arrowhead: Binary fission; Black arrow: Rod–shaped bacteria; Yellow arrow: Multi–layered membrane–bound compartment; Asterisk (*): Digested bacterial cell, Endo: Endo–cyst wall; Ecto: Ecto–cyst wall. Scale bars, A: 1μm, B: 500nm, C and D: 2μm.

Under transmission electron microscopy, AK1–PA bacteria exhibited a rod–shaped morphology, enclosed within multi–layered phagolysosome like structure (**Fig 5**). A distinct phagolysosomal membrane was evident, encapsulating the engulfed bacteria. No intranuclear stage was identified but a few cells were observed in close proximity to the nuclear membrane. Within the phagolysosome, it was intriguing to observe transverse bacterial cell division through binary fission (**Fig 5B**). Both undigested and digested bacterial cells were found within the same phagocytic vacuole as intact and disintegrated with granules, respectively. In the cystic stage of the host AK1, a bacteria–like structure was detected close to endo–cyst wall (**Fig 5C–5D**).

### 3.4. AlkDala–labelling confirms the presence of metabolically active *P*. *aeruginosa* within the amoebal host

**Fig 6:**
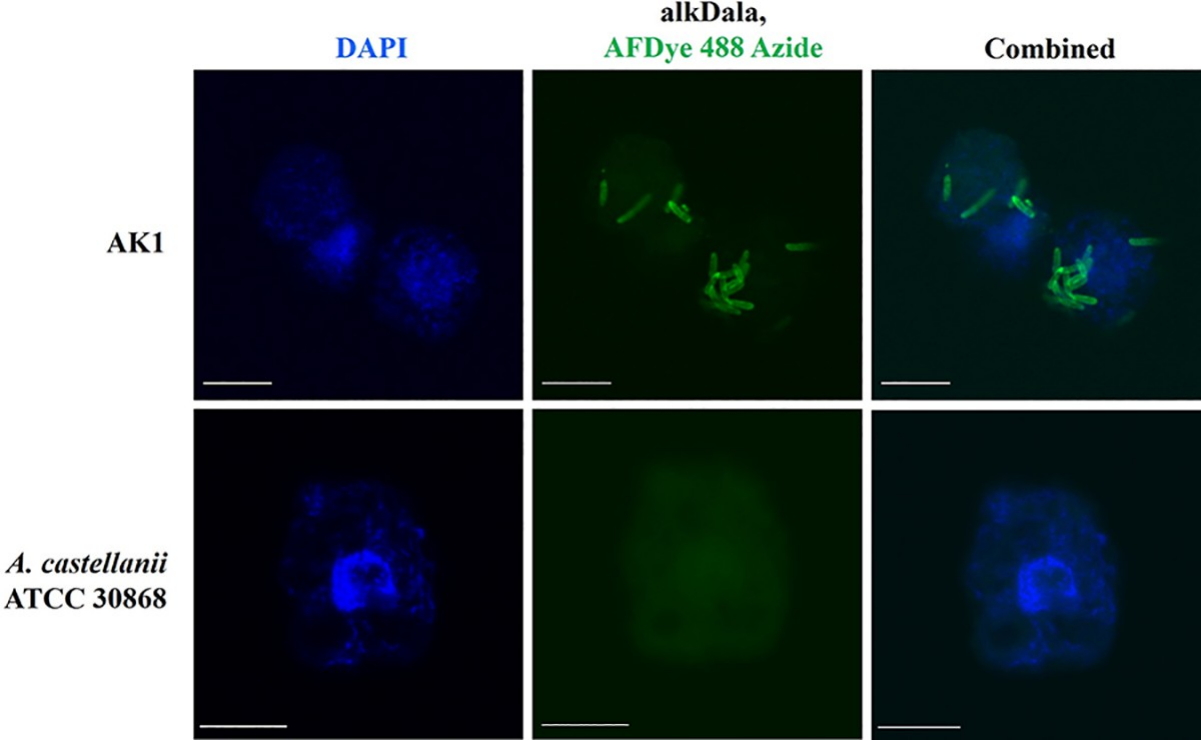
Alkyne–functionalized D–alanine (AlkDala) labels metabolically active bacteria in *Acanthamoeba* trophozoites. AlkDala labelling was tested on AK1 trophozoites (first panel) and the green fluorescence indicates metabolically viable intracellular bacteria. *A*. *castellanii* (ATCC 30868) was used as a control (second panel) and DAPI was used to stain host nucleus. Scale bars, 10μm (first panel) and 15μm (second panel).

To confirm the viability of intracellular *P*. *aeruginosa* (AK1–PA) cells within trophozoites, we used (R)–α–Propargylglycine as a D–alanine analogue in the assay to incorporate into the bacterial cell’s peptidoglycan during cell wall biosynthesis. Subsequently, the cells were labelled with a fluorescent Azide probe using click chemistry. Prior to use with *Acanthamoeba* cells, alkDala–labelling was tested for specificity and efficacy with PAO1 and *S*. *aureus* (SA32, clinical isolate) using both viable and heat killed bacteria. As expected, heat–killed bacteria or bacteria treated only with D–alanine (without alkyne group) did not label. *A*. *castellanii* ATCC 30868, devoid of any intracellular bacteria was used as a control. The confocal microscopy showed alkDala–labelled bacterial cells within the amoebal host indicating the presence of metabolically active bacteria harboured by *Acanthamoeba* strain AK1 (**Fig 6**). These results are consistent with the electron microscopy observation where bacterial cells undergoing binary fission were seen indicating the presence of live *P*. *aeruginosa* residing within the host cell.

### 3.5. Effect of *Acanthamoeba*–adaption on *exoU* positive *P*. *aeruginosa* motility

The intracellular *P*. *aeruginosa* AK1–PA possessed *exoU* but not *exoS* (S6 Fig). Two open reading frames (ORFs) were identified in the 1176–bp sequence of *exoU*. The amino acid sequence coded by ORF1 (322→1071, 249 aa) showed a high similarity with the T3SS effector putative cytotoxic *exoU* in blastp research. A few single–nucleotide polymorphisms [SNPs; V1056C (GGC→GCG), V1084G (CAA→CGA), V1090G (CAA→CGA), V1101T (GAA→GTA), V1113G (CCA→CGA), and V1121T (CAA→CTA)] and deletion mutations [Δ1 bp (12), Δ1 bp (1028), Δ1 bp (1092), Δ1 bp (1103), Δ1 bp (1126), Δ1 bp (1157), and Δ1 bp (1168)] were identified when the sequence of *exoU* of AK1–PA (1176 bp sequence) was compared with the *exoU* genes of other *P*. *aeruginosa* strains deposited in NCBI. However, high–throughput whole genome sequencing is required to confirm the SNPs and deletions.

**Fig 7:**
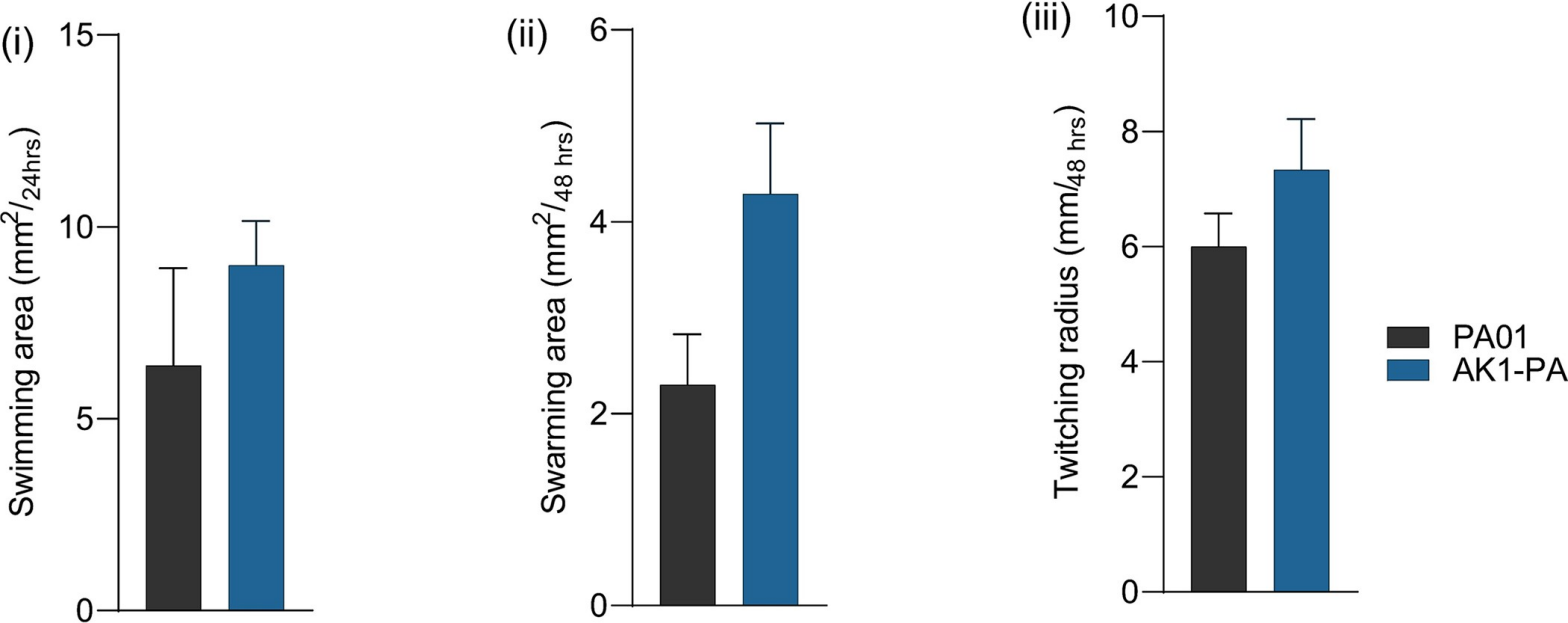
Effect of *Acanthamoeba* adaptation on the motility of intracellular *P*. *aeruginosa* (AK1–PA) compared with PA01; (**i**) swimming, (**ii**), swarming, and (**iii**) twitching. Data are mean ± SEM. The mean motility data were analysed using an unpaired *t* test (all *p*–values >0.05).

*Acanthamoeba* adapted *P*. *aeruginosa* AK1–PA showed slightly greater swimming motility compared to the non–adapted wild–type strain PA01, but the difference was not significant (*p*>0.05, **Fig 7i**). The mean swarming motility of AK1–PA strain was about double that of the PA01, but the difference was not significant (*p*>0.05, **Fig 7ii**). Similarly, the twitching distance (radius) exhibited by AK1–PA was 1.3mm higher compared to the PA01, but again this was not significant (*p*>0.05, **Fig 7iii**).

### 3.6. *Acanthamoeba* adapted *P*. *aeruginosa* AK1–PA showed enhanced intracellular survival within human macrophages

**Fig 8:**
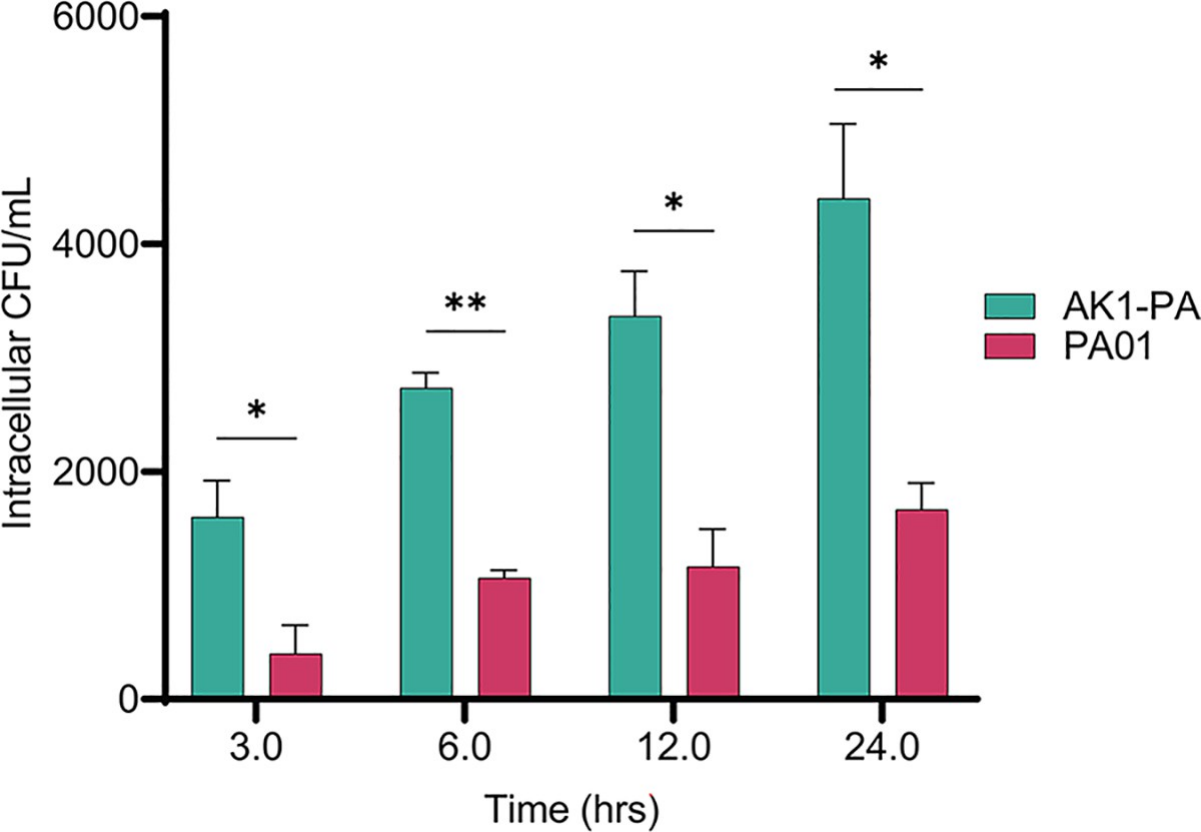
To assess the intra–vacuolar replication of *P*. *aeruginosa* strains (AK1–PA and PA01) within hMDMs monolayers, infected hMDMs monolayers were lysed at 3, 6, 12, and 24 h p.i. and serial dilutions were plated on agar plates to quantify the colony–forming units (CFUs). The data represents the mean CFUs ± SEM from three independent experiments (*n* = 3). Student’s t test was used to compare the intracellular numbers of AK1–PA and PA01 at different time points p.i. (*, *p*<0.05; **, *p*<0.01; ***, *p*<0.001).

To assess whether the intracellular survival of *Acanthamoeba*–adapted bacteria extends to other higher eukaryotic phagocytic cell, the survival abilities of adapted AK1–PA and non–adapted *P*. *aeruginosa* PA01 were compared using primary human macrophages (hMDMs) (S7 Fig). At 3 hrs post–infection, the AK1–PA strain infected macrophages had more than 4–fold greater numbers of intracellular bacteria compared to the wild–type strain (**Fig 8**, *p*<0.05). The difference was approximately 3–fold at 24 hrs p.i. (*p*<0.05).

**Fig 9:**
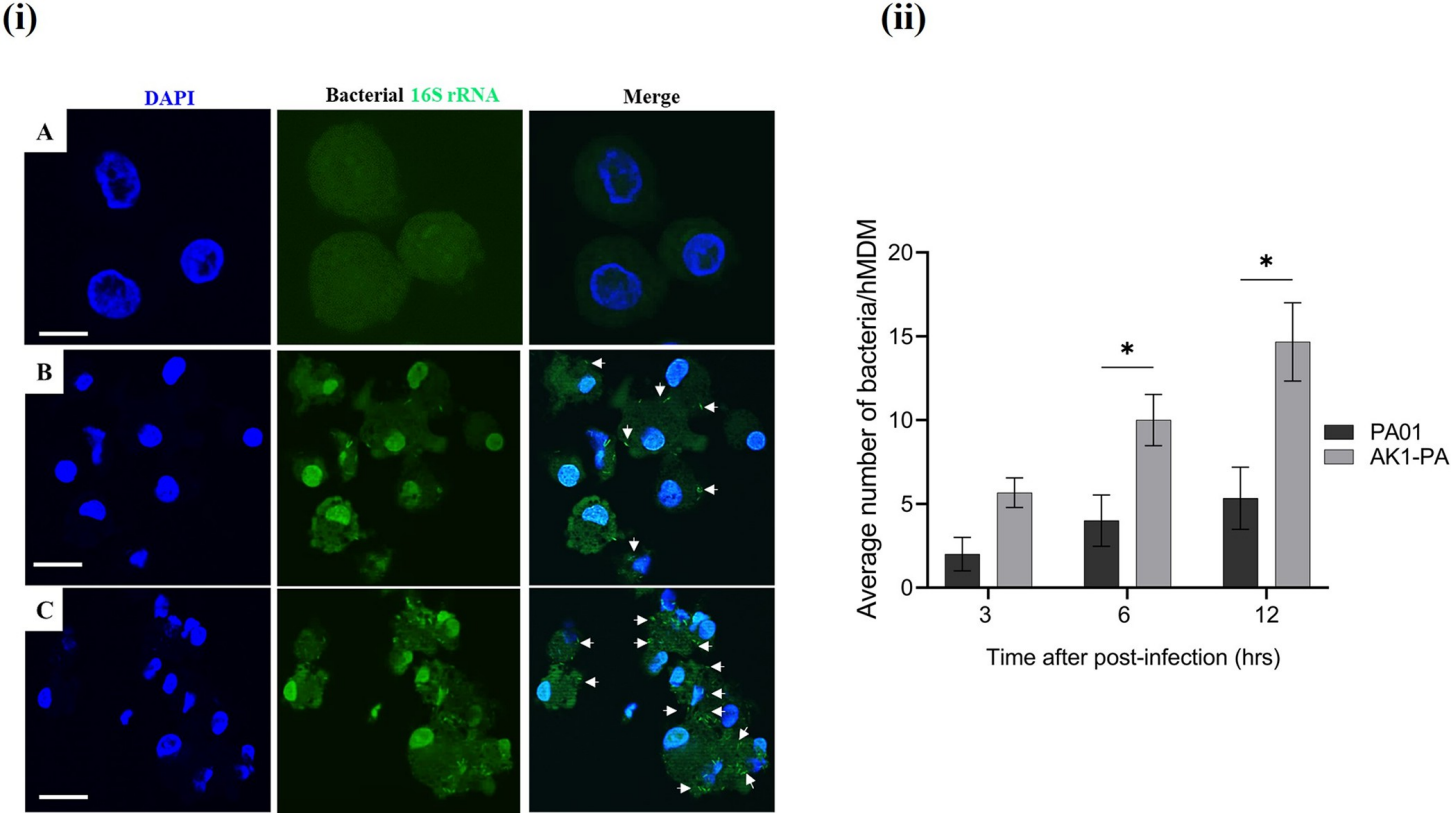
Representative confocal microscopy images of hybridization assay (i) of hMDMs cells after three hours post–infection; **A**. mock–infected cells, **B**. infected with wild–type (PA01), and **C**. infected with *P*. *aeruginosa* (AK1–PA). Compared to uninfected cells, the structure of macrophage cells infected with AK1–PA exhibited a slight disorganization. Bacterial numbers in hMDMs cells were enumerated by confocal microscopy (**ii**). Student’s t test was used to compare the number of AK1–PA/hMDM versus wild–type PA01 at 3, 6, and 12 hrs p.i. White arrow shows bacteria within macrophage cells. Scale bar represents 20μm.

EUB338 probe labelling also revealed a higher number of *P*. *aeruginosa* AK1–PA in macrophage cells compared to the wild–type strain 3 hrs p.i. (**Fig 9i**), indicating enhanced intracellular proliferation of *Acanthamoeba*–adapted *P*. *aeruginosa* in human immune cells. Uninfected and PA01–infected macrophage cells showed normal morphology, while those infected with AK1–PA were slightly disorganized (**Fig 9i, C**). These results suggest that amoeba–adapted bacteria were more toxic to the macrophages than the non–adapted strain. To confirm the intracellular presence of bacteria at the single–cell level, we enumerated bacterial load per hMDM cell at 3–12 hrs post–infection. At 3 hrs p.i., we observed approximately 3–fold more bacteria per hMDM infected by AK1–PA compared to PA01, but this difference was not significant (*p*>0.05) (**Fig 9ii)**. However, at 6 and 12 hrs post–infection, the bacterial load in macrophage cells harbouring AK1–PA strain was significantly higher than that of the wild–type strain (*p*<0.05).

### 3.7. The *Acanthamoeba* strain with intracellular bacteria induced acute keratitis

The study investigated whether naturally acquired intracellular bacteria play a role in inducing severe *Acanthamoeba* keratitis in rats’ eyes. In the first phase, axenically cultured *Acanthamoeba* cells with (AK1) and without (AK11) intracellular bacteria were trans–corneally inoculated into the rat’s cornea and the progression of infection was observed microscopically (**Fig 3**). Hybridization assay has shown approximately four bacteria in each trophozoite of the AK1 strain, so 4×10^4^ *P*. *aeruginosa* bacteria were inoculated when 10^4^ trophozoites were delivered into each eye of group B rats, while group A received only 10^4^ trophozoites (AK11). The clinical features of AK were assessed, recorded, and graded using slit lamp examinations from day 1 to 5 post–infection. Group A rats cornea inoculated with *Acanthamoeba* devoid of any intracellular bacteria, showed a few focal infiltrates on day 4 of infection. The corneas remained transparent during the infection period with very mild keratitis showing no signs of inflammation (**Fig 9i**).

**Fig 10:**
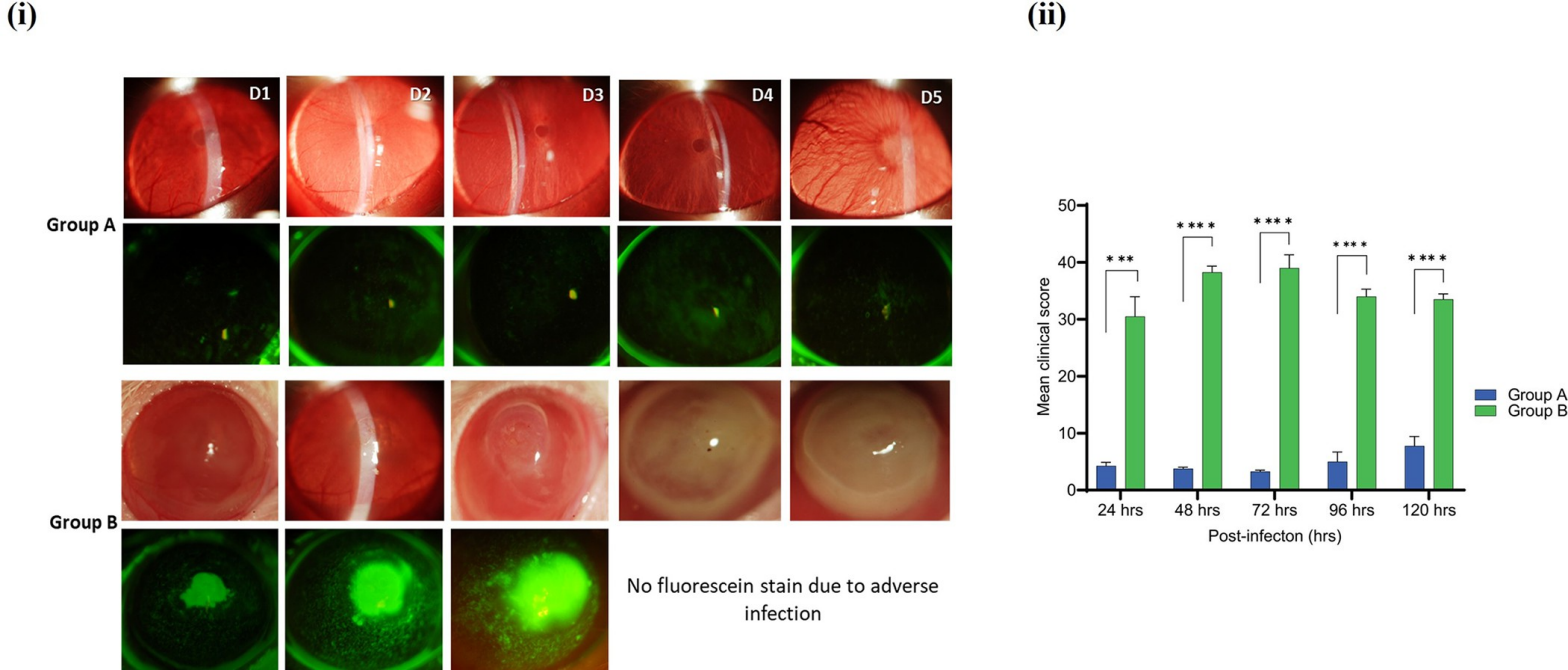
Representative slit lamp photographs of experimentally induced *Acanthamoeba* keratitis in rats’ right eyes caused by *Acanthamoeba* alone (group A) or *Acanthamoeba* with viable *P*. *aeruginosa* (AK1) (group B) from day 1 to 5 p.i. The first panel of each group represents bright field, and the second shows a fluorescein–stained micrographs (**i**). The mean clinical score of group A and B rats was compared using an unpaired *t*–test (**ii**) (*, *p*≤0.05, ***, *p*≤0.001, ****, *p*≤0.0001, *n* = 4 in each group).

In contrast, group B rats infected with an *Acanthamoeba* strain containing viable intracellular *P*. *aeruginosa* exhibited severe infection with a large ring infiltrate in the centre of the cornea within 48 hrs p.i. All rats of group B rapidly developed keratitis with anterior chamber inflammation, severe conjunctivitis, diffuse infiltrates, and mild corneal edema. By day 4, the ocular lesions had progressed to corneal epithelial ulceration, accompanied by extensive stromal inflammation. Fluorescein staining of the cornea was not performed for group B rats on days 4 and 5 due to acute infection (**Fig 10i**). Blood vessels and random superficial lesions were developed with an extensive zone of corneal opacity and necrosis at the centre on day 4 in group B rats. Based on slit lamp examination, infection was at its peak on day 3 in group B rats, but it remained mild even on day 5 in group A rats. The mean clinical scores of group A and group B rats revealed a significant difference on each day of post–inoculation (**Fig 10ii**). Five days after infection, rats were euthanised due to acute keratitis, which was the clinical end point. The weights of both groups rats remained normal compared to starting weight (S8 Fig).

**Fig 11:**
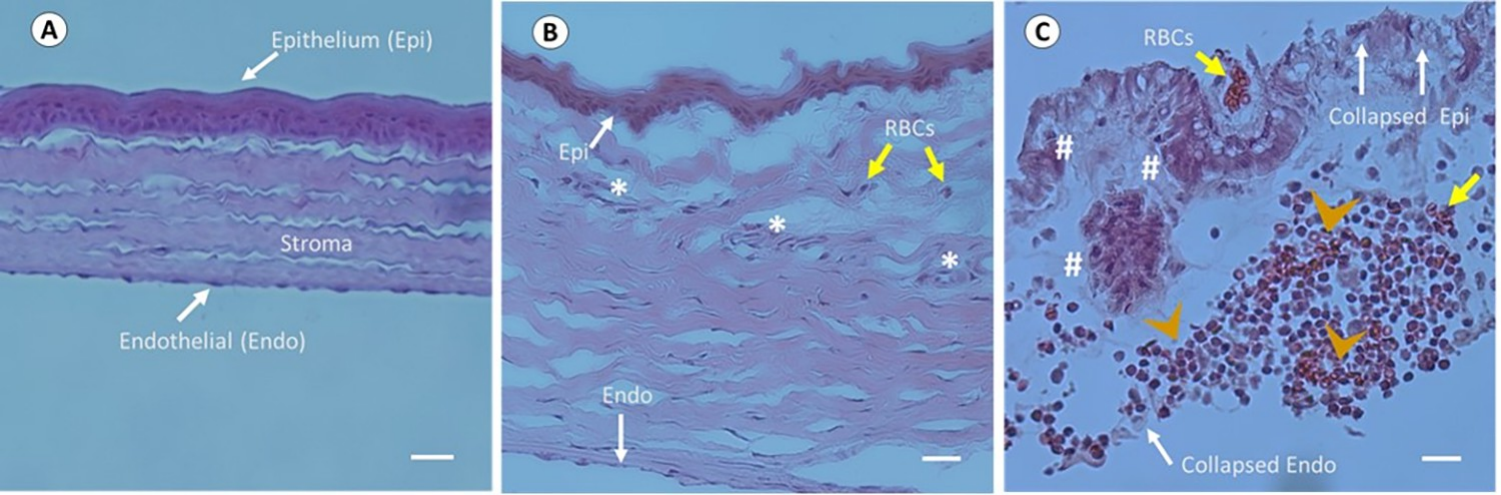
Histological observation of AK induced in Wister rats by intrastromal injection of amoebal trophozoites. Five μm thick corneal section of the uninfected left eye stained with HE exhibited well–defined epithelium and stroma without any infiltration of inflammatory cells (**A**), but a portion of endothelium was fell off during micro–sectioning in the cryostat. Corneas infected by *Acanthamoeba* alone (group A) showed a few areas of necrosis and blood cells with the epithelium and endothelium slightly disorganized (**B**). In group B, the stromal region infected by *Acanthamoeba* with intracellular bacteria exhibited haemorrhagic necrosis with fibrin deposition, desquamated cells, inflammatory infiltrate, and a collapse of the corneal epithelium and stromal structure (**C**). Indicators: White arrow, corneal epithelium, and endothelium; Yellow arrow: RBCs; Asterisk (*): Necrosis; Hash (#): Haemorrhagic necrosis; and Arrowhead: Inflammatory infiltrate. Scale bar represents 30μm for A, 20μm for B and C.

The HE staining of corneal microsections from the control left eyes had a normal appearance showing a well–defined stromal structure with regularly arranged stromal fibres and the absence of any changes in the corneal epithelium (**Fig 11A**). However, the corneal epithelium and endothelium displayed areas of necrosis in the stromal region of group A rats (**Fig 11B**). In contrast, histological analysis showed the inflammatory infiltrate at centre of stroma, and the epithelium and endothelium of cornea were completely collapsed in group B rats, with haemorrhagic necrosis and desquamated cells throughout the cornea (**Fig 11C**). Furthermore, the observation showed substantial quantities of cellular debris and accumulated fibrin, neovascularisation surrounded by infiltrate leukocytes and profusion of granulation tissue.

**Fig 12:**
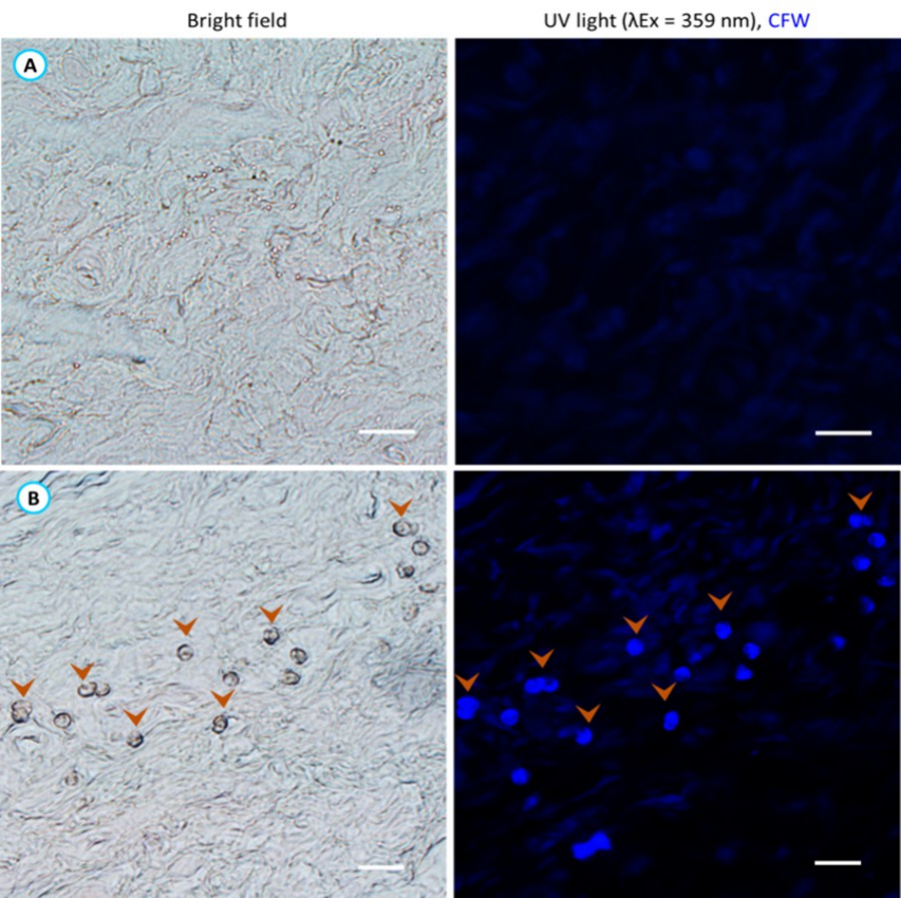
Thin (10μm) corneal sections from group A (**A**) and B (**B**) rats were stained with CFW. Corneal sections from group B exhibited blue fluorescence with cyst–like structures resembling amoebic cysts, while no staining was observed in group A. Arrowhead indicates *Acanthamoeba* cysts like structures under light and fluorescence microscopes. Scale bar represents 20μm.

Cyst–like structures were observed in the corneal sections of group B rats when examined under a light microscope. Therefore, calcofluor white stain (CFW), a chemo–fluorescent dye that binds with cellulose in the cyst cell wall was used to stain suspected *Acanthamoeba* cysts. Interestingly, those suspected cyst–like structures in the corneal sections of group B rats exhibited a bluish white colouration under a fluorescence microscope (**Fig 12**). The corneal sections of group A rats showed no CFW staining.

To confirm cells stained with CFW were *Acanthamoeba* cysts, suspected cells (~150 cells) were precisely collected from heterogeneous cell population from corneal sections of group B rats. Corneal sections were transferred onto a PEN (polyethylene naphthalate) membrane slide (Carl Zeiss Microscopy, GmbH, Germany), and cyst–like cells were selected, excised, and collected using non–contact laser pressure catapult (LPC) procedure. The catapulted cells were collected in a collection tube and gDNA was extracted followed by PCR using the *Acanthamoeba* specific JDP primer pair. PCR confirmed that the cyst–like cells labelled with CFW in the corneas of group B rats were *Acanthamoeba* cysts (S9 Fig).

**Fig 13:**
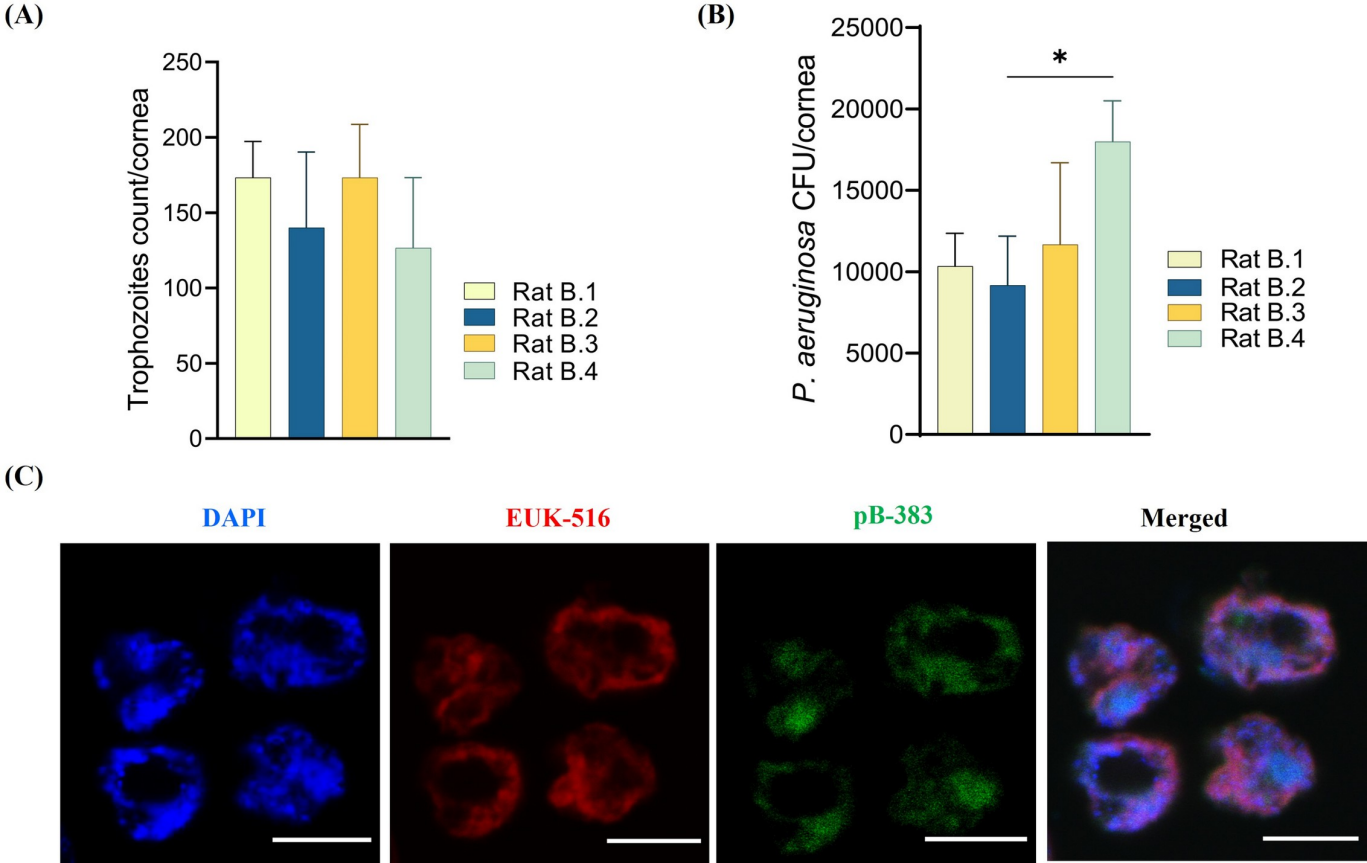
The mean trophozoites and *P*. *aeruginosa* counts in each corneal homogenate of group B rats’ eyes (**A, B**). *Acanthamoeba* trophozoites recovered from the corneal homogenate of group B rats (AK10) were used for FISH assay to assess presence of originally acquired intracellular *P*. *aeruginosa* using the pB–383 probe. Representative FISH images demonstrate the absence of intracellular bacteria in any of the amoebal cells within the population (**C**). Scale bar represents 10μm.

Corneal homogenates were cultured from the infected right eyes of both group rats to re–isolate intrastromally inoculated *Acanthamoeba* from group A and *Acanthamoeba* along with intracellular *P*. *aeruginosa* from group B. Non–nutrient agar (NNA) and TSA were used to culture *Acanthamoeba* and intracellular *P*. *aeruginosa* from the homogenates, respectively. No growth was observed from any of the group A homogenates while *Acanthamoeba* trophozoites and *P*. *aeruginosa* colonies were grown from group B. NNA plates were cultured for three weeks at 32°C and trophozoites number were counted using an inverted microscope (IX71, Olympus America, NY, USA). Among the four corneal tissues of group B, the trophozoites count was not significantly different in the cornea six days after infection (*p*>0.05). Trophozoites recovered from corneal tissues were approximately 18 to 25–fold lower compared to the original inoculum (10^4^/cornea) but the mean count across four corneal tissues was not significantly different (*p*>0.05) (**Fig 13A**). Similarly, *P*. *aeruginosa* counts ranged from 9.2x10^4^ to 1.8x10^4^/cornea, and the counts were not significantly different (*p*>0.05) except between rat B.2 and B.4 (*p* = 0.04) (**Fig 13B**). *Acanthamoeba* trophozoites (AK10) recovered from group B rats’ corneal tissue appeared to have expelled all their intracellular *P*. *aeruginosa* during infection in rat’s eye as no intracellular bacteria seen in hybridization assay (**Fig 13C**). *Acanthamoeba* (AK10) and *P*. *aeruginosa* re–isolated from the corneal tissues of group B rats were utilised in the second phase of experimental AK study to investigate whether the severe keratitis observed in group B was attributed to the co–presence of both *Acanthamoeba* and intracellular bacteria.

**Fig 14:**
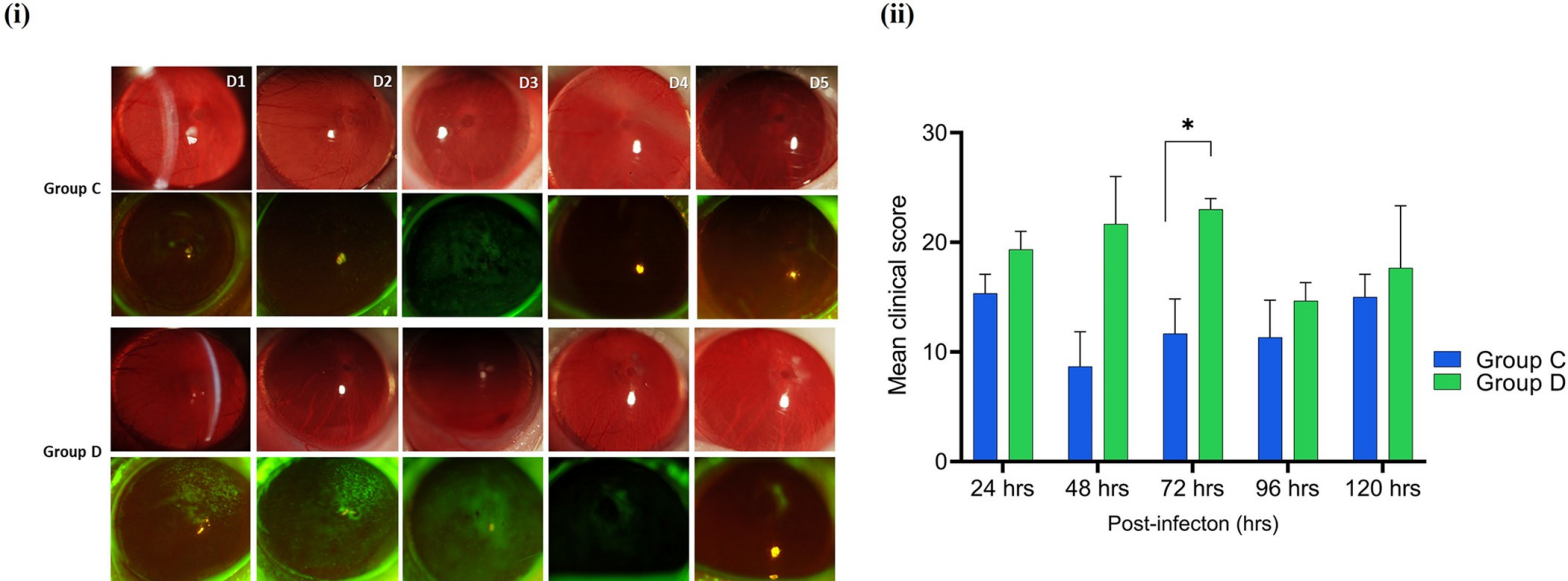
Clinical photographs of rats’ right eyes with experimentally induced *Acanthamoeba* keratitis. Development of keratitis in rats’ corneas inoculated only with *Acanthamoeba* (AK10) (group C) or *P*. *aeruginosa* (group D) from day 1 to 5 p.i. Representative slit lamp photographs (**i**), before (first panel of each group) and after (second panel of each group) application of fluorescein stain. The mean clinical score of group C and D rats was compared using an unpaired *t*–test (**ii**) (*, *p*≤0.05, ***, *p*≤0.001, ****, *p*≤0.0001, *n* = 3 in each group).

In the second phase, *Acanthamoeba* (AK10) was inoculated into group C (*n* = 3) rats, while *P*. *aeruginosa* recovered from corneal tissues was used to induce keratitis in group D (*n* = 3) rats, following the same procedure as that used in phase I. *Acanthamoeba* (AK1) contained an average of four bacterial cells per trophozoite. Therefore, a total of 4x10^4^ *P*. *aeruginosa* cells were inoculated into the group D rats to achieve a bacterial load similar to that of group B rats. In group C rats, minor focal and diffuse infiltrates along with faint linear epithelial corneal opacities were noted 72 hrs p.i. In contrast, group D rats exhibited moderate conjunctivitis, diffuse infiltrates, slight corneal opacity, and diffuse central edema. Retained fluorescein stain was also clearly visible 48 hrs p.i. (**Fig 14i**). Clinical corneal lesions were similar between all rats in group D with the infection reaching its peak on day 3, which was significantly higher compared to group C (**Fig 14ii**). Between p.i. days 4 and 5, corneal opacities and infection slowly decreased in group B, but the infection remained almost similar in group C characterised by focal and diffuse infiltrates at the centre of cornea. Consistent to slit lamp observation, HE staining of corneal microsections from group C rats showed a discrete accumulation of red blood cells (RBCs) accompanied by slight epithelial disorganization. No evidence of inflammatory infiltrates or severe necrosis was detected. Necrosis–like structures with RBCs, desquamated cells, mild inflammatory infiltrate, and notable disorganization of both the corneal epithelium and stromal structure were seen in corneal tissues of group D rats (S10 Fig). Similar to phase I, the weights of rats in both groups did not change significantly compared to their starting weights (S11 Fig.

**Fig 15:**
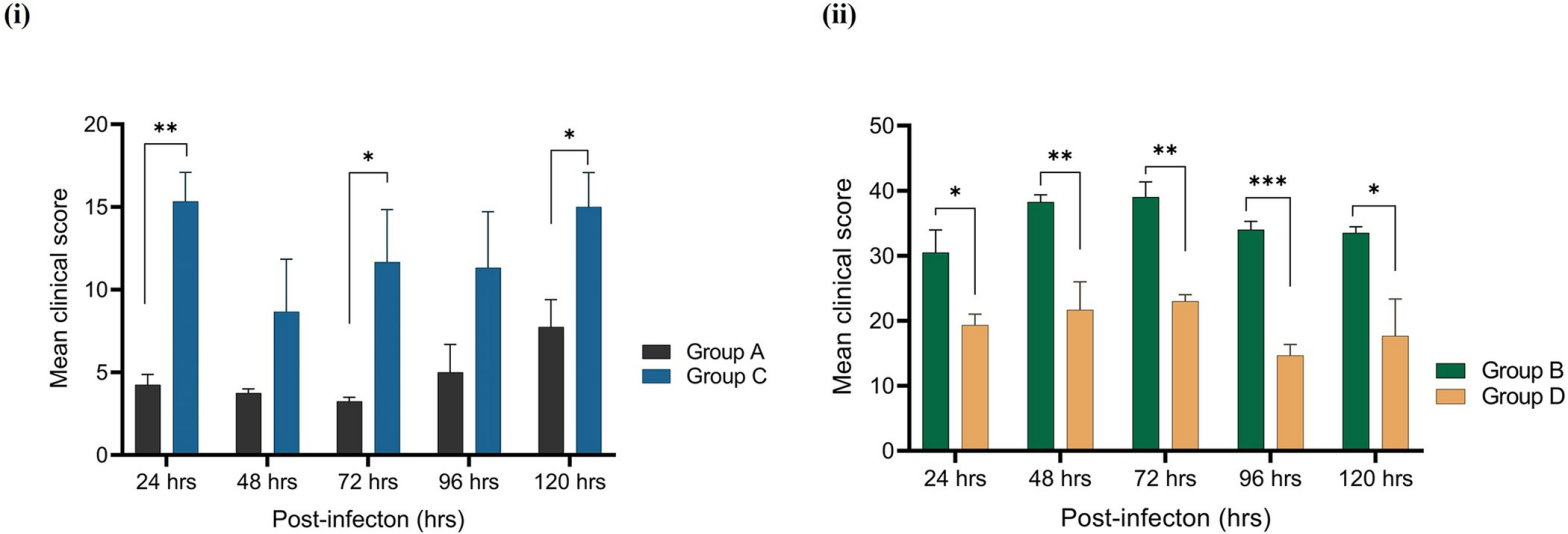
Mean keratitis clinical scores (±SEMs) were calculated every 24 hrs for rats with experimentally induced *Acanthamoeba* keratitis. Group A was infected with *Acanthamoeba* strain (AK11) isolated from an AK patient, group C was infected with *Acanthamoeba* strain (AK10) re–isolated from rat’s corneal tissue (**i**), group B was inoculated *Acanthamoeba* carrying intracellular *P*. *aeruginosa*, and group D received *P*. *aeruginosa* alone that was recovered from tissue of group B rats (**ii**). Groups were compared using an unpaired *t* test. (*, *p*≤0.05, ***, *p*≤0.001, ****, *p*≤0.0001).

We also compared the mean clinical scores of group A rats with those of group C rats, which were infected by different strains of *Acanthamoeba* devoid of any intracellular bacteria. The average clinical score of group C rats was significantly higher (*p*<0.05) than that of group A rats at 24, 72, 96, and 120 hrs p.i. (**Fig 15i**). This indicates that *Acanthamoeba* may have become more virulent after adapting to the rat cornea, as group C rats were infected with *Acanthamoeba* re–isolated from corneal tissue. Similarly, infection severity was significantly higher (*p*<0.05) in presence of *Acanthamoeba* with naturally acquired intracellular *P*. *aeruginosa* (group B) compared to cases where only intracellular *P*. *aeruginosa* was inoculated (group D) at all time points from day 1 to 5 p.i. (**Fig 15ii**).

### 3.8. Keratitis induced in rats by *Acanthamoeba* with intracellular bacteria increase stromal neutrophils

**Fig 16:**
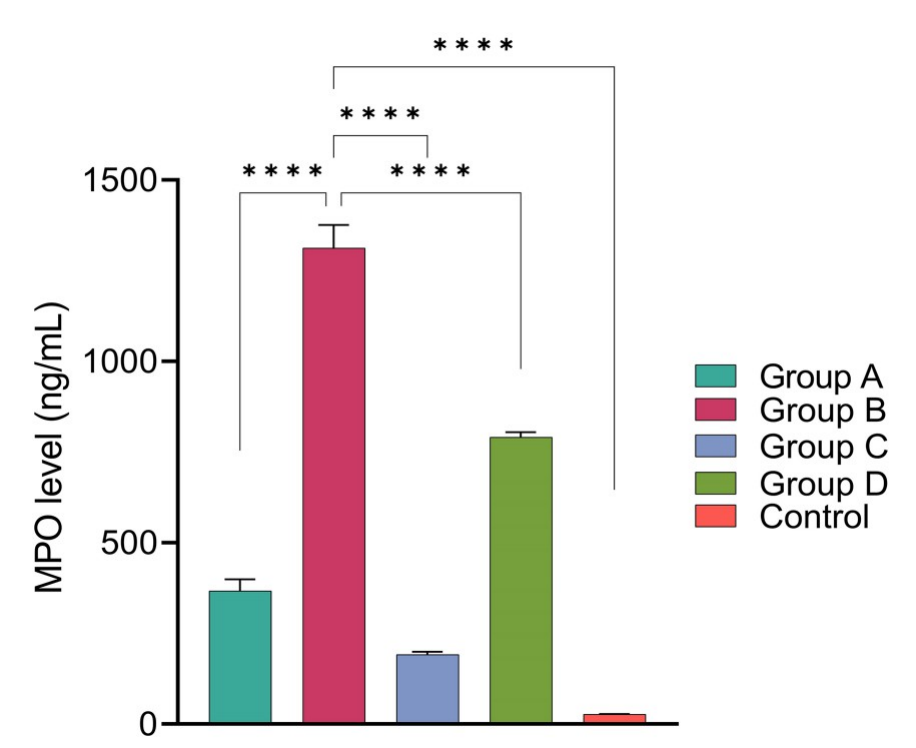
Relative myeloperoxidase (MPO) concentration measured in ng/mL at 6 days post–challenge with *Acanthamoeba* alone (AK11) (group A), *Acanthamoeba* with intracellular *P*. *aeruginosa* (AK1) (group B), *Acanthamoeba* (AK10) (group C) and *P*. *aeruginosa* (group D) recovered from corneal tissue of group B rats. All values are mean ± SEM (*n* = 3), one–way ANOVA, followed by Tukey’s multiple comparison test for intergroup comparisons; post hoc test, was performed (*, *p*≤0.05, ***, *p*≤0.001, ****, *p*≤0.0001).

The estimation of relative neutrophil numbers in the corneas was performed using a myeloperoxidase assay. As expected, high level of MPO expression was observed in group B rats (1312.3 ng/mL) infected with *Acanthamoeba* containing naturally acquired intracellular *P*. *aeruginosa* (**Fig 16**). Group D rats, which were infected with *P*. *aeruginosa* re–isolated from the corneal homogenate of group B rats, showed an MPO level approximately half that of group B (791.3 ng/mL). However, MPO levels were significantly lower in rats infected by *Acanthamoeba* alone as observed in group A (367.5 ng/mL) and group C (191.6 ng/mL) but were significantly higher (*p*<0.05) when compared to the uninfected left eye. We compared the MPO concentration of group B with that of groups A, C, and D which was significantly different (*p*<0.05).

## 4. Discussion

In this study, an AK model was established in rats using trans–corneal inoculation of *Acanthamoeba* suspension. While contact lens–acquired infection is likely predominantly an epithelial infection in its initial stages, it progresses to a stromal infection in later stages. Therefore, AK induced by inoculating an amoebal suspension into the stromal region represents the advanced stage of AK infection [44]. To our knowledge, this is the first study to investigate the role of naturally acquired live intracellular bacteria in the development of AK *in vivo*, which we examined in two phases using two recent isolates of *Acanthamoeba* strains from AK patient corneal specimen and domestic tap water.

This study results showed the viability of naturally acquired intracellular *P*. *aeruginosa* in *Acanthamoeba*. This *P*. *aeruginosa* had the ability to multiply within the host cells, as evidenced by binary fission observed under electron microscopy. Consistent with our study, previous studies have also observed intracellular bacterial cells undergoing binary fission within amoebal host [1,45]. *P*. *aeruginosa* was highly motile with swimming motility, and possessed the *exoU* gene. Some SNPs and deletion mutations were identified in the *exoU* gene short sequence, but high throughput sequencing is necessary to confirm the presence of mutations. A previous study [46] has reported that adaptation to the *Acanthamoeba* host leads to *P*. *aeruginosa* strains with attenuated virulence during *in vitro* co–incubation. However, long–term amoeba adapted *P*. *aeruginosa* showed enhanced survival in murine macrophage and human neutrophils [46]. In another study, intra–amoebal host adaptation of *Vibrio cholerae* drove the selection of virulence associated traits that resulted in enhanced colonisation in zebrafish [47]. Similar findings were observed in our study, indicating that intracellular *P*. *aeruginosa* showed significantly higher survival rates in hMDMs compared to the non–adapted wild–type *P*. *aeruginosa*. *Acanthamoeba* adapted *P*. *aeruginosa* strain exhibited rapid swimming and chemotaxis, making it more readily taken up by macrophages. Amoebal trophozoite is often compared to human macrophage due to shared similarities in molecular functions and phagocytosis [48]. Interestingly, some of the bacteria utilise the same genes for intracellular replication within human macrophages and *Acanthamoeba* [49,50]. The ability of endosymbiotic bacteria to resist digestion by trophozoites in response to environmental predatory pressure may drive the evolution of traits that may help pathogens to survive during phagocytosis by higher eukaryotic cells [47,51]. Future studies will be necessary to gain a deeper understanding of how bacteria by evading amoebal phagocytosis enhance their capability to survive in human immune cells [52,53].

We observed severe keratitis in rats when *Acanthamoeba* with naturally acquired *P*. *aeruginosa* was inoculated intrastromally compared to rats that only received *Acanthamoeba*. *Acanthamoeba* strain with no intracellular bacteria resulted in minimal keratitis with no observable signs of inflammation and the corneas remained clear during five days of post–inoculation. This observation is consistent with earlier findings which have reported that *Acanthamoeba* by itself, without the presence of bacteria did not lead to keratitis in *in vivo* [26,54]. But some other studies have suggested that *Acanthamoeba* alone can initiate the corneal infection and can be pathogenic in animal models [55–57]. However, it is important to note that the potential presence of amoebic bacterial endosymbionts was not assessed in those studies.

Intracellular bacteria found in *Acanthamoeba* can exacerbate corneal epithelial damage as has been observed in AK patients [20,25] and cell models [20,23]. Release of bacterial endosymbionts in a compromised cornea may boost inflammation and may worsen the clinical outcomes [20, 58]. The underlying mechanism behind the enhanced cytopathogenicity linked to bacterial endosymbionts remains unclear. However, it is intriguing to consider the possibility that molecules, such as mannosylated glycoproteins in the bacteria cell wall could trigger the release of cytotoxic serine and metalloproteases by the trophozoites [59]. In turn, these proteases might perform in concert to produce a cytopathic effect leading to severe keratitis. The inflammatory response is likely to be instigated by the intracellular contents of acanthamoebae [60]. Concealed endosymbionts within acanthamoebae might constitute intracellular antigens, potentially driving increased inflammation and corneal cell damage during AK. In addition, accelerated growth and enhanced motility of *Acanthamoeba* were observed in the presence of bacterial endosymbionts *in vitro* [61]. This phenomenon may also occur in the cornea, where endosymbionts could serve as a nutrient source for the amoebal host further supporting *Acanthamoeba* proliferation.

Bacterial endosymbionts can maintain a stable interaction with *Acanthamoeba*, but its transferability to new host cells might be limited [62]. In the current study, *Acanthamoeba* trophozoites previously containing *P*. *aeruginosa* were recovered from infected corneal tissue without their bacterial cargo. It appeared that trophozoites expelled their naturally harboured intracellular bacteria in rats’ cornea during keratitis, as no intracellular bacteria was seen in trophozoites re–isolated after infection. This indicates that amoebal host can release undigested intracellular bacteria into the cornea, potentially leading to severe polymicrobial keratitis involving both amoebae and bacteria. We also observed reduced (18 to 25–fold) trophozoite load in corneal tissues after 5 days p.i. compared to the initial inoculum. This reduction could potentially be attributed to the rapid growth of released bacteria. All rats infected with *Acanthamoeba* containing endosymbiont exhibited acute keratitis accompanied by severe conjunctivitis, diffuse infiltrate, and extensive stromal inflammation within 48 hrs p.i. Histopathological analysis revealed complete collapse of corneal epithelium and endothelium with inflammatory infiltrates, significant cellular debris, accumulated fibrin, and neovascularization surrounded by infiltrating leukocytes. Furthermore, an increased number of neutrophils in the corneal homogenates of rats with acute AK was observed. However, rats infected only with *Acanthamoeba* exhibited only few areas of necrosis in the stromal region in comparison. In a separate study, pre–treatment of virulent *Acanthamoeba* isolates with antibiotics to eliminate their bacterial endosymbionts led to the loss of pathogenicity in rabbits [54]. These findings suggest that bacteria could be a potential prerequisite for *Acanthamoeba* to initiate *in vivo* keratitis. Endosymbiotic bacteria of *Acanthamoeba* often shift to a viable but non–culturable (VBNC) state, while in this study, we found live and culturable *P*. *aeruginosa* within the amoebal host. *In vitro* observations have shown that *Acanthamoeba* can expel undigested bacteria even in the form of expelled food vacuoles (EFVs), allowing several hundred bacteria to escape from these EFVs leading to increased infectivity *in vivo* [63].

To confirm whether acute keratitis was due to amoebal host or released intracellular *P*. *aeruginosa*, we inoculated them separately in two different groups of rats. Notable keratitis with mild corneal opacity and conjunctivitis developed in rats infected with intracellular *P*. *aeruginosa* 48 hrs p.i. However, the mean clinical score was significantly lower than that of rats infected with *Acanthamoeba* containing intracellular *P*. *aeruginosa*. Consistent to our study, Nakagawa et al. (2017) have noted high clinical scores in corneas inoculated with *P*. *aeruginosa* engulfed *Acanthamoeba* compared to *P*. *aeruginosa* alone [26]. To maintain the same bacterial load as that of naturally acquired intracellular bacteria, we inoculated a total of 4x10^4^ *P*. *aeruginosa* per cornea which is lower compared to other experimental studies [64,65]. Therefore, the infection was not as acute as would be expected in an induced *Pseudomonas* keratitis model [66]. Conversely, minor focal and diffuse infiltrates with a transparent cornea were observed in rats infected with *Acanthamoeba* alone 72 hrs p.i., resembling the first phase of *Acanthamoeba* infection without any bacteria within. Hence, we propose that the simultaneous infection of *Acanthamoeba* along with released intracellular bacteria could be a major contributing factor to the progression of severe AK [26]. With the increasing prevalence of coinfections among AK patients [18,19,21], which often lead to severe outcomes, this study contributes to our understating of the role of intracellular bacteria and potential bacterial endosymbionts in the development of acute *Acanthamoeba* keratitis. The findings presented in this study showed that coinfections in *Acanthamoeba* keratitis are potentially attributed to the carriage of bacteria by *Acanthamoeba*. Thus, it is worth investigating the possible presence of intracellular bacteria in corneal isolates of *Acanthamoeba* during routine culture of corneal specimens for *Acanthamoeba* growth. After confirming the presence of intracellular bacteria in the corneal isolate of *Acanthamoeba*, considering the addition of suitable antibiotics as an adjuvant to standard antiamoebic therapy (AAT) may be beneficial in mitigating the virulence-enhancing traits of intracellular bacteria [58].

## 5. Conclusion

This study represents the first experiment to investigate the role of naturally acquired viable intracellular *P*. *aeruginosa* in the development of *Acanthamoeba* keratitis *in vivo*. Identification of live *P*. *aeruginosa* within *Acanthamoeba* confirms the existence of a stable interaction between intracellular bacteria and the amoebal host with limited transferability. This study adds new data confirming previously suspected intracellular survival of bacteria in amoebal host, which can lead to enhanced survival in human immune cells. The experimentally induced AK in rats’ cornea suggests that the presence of bacteria could be a potential prerequisite for *Acanthamoeba* to develop acute keratitis *in vivo*. As *P*. *aeruginosa* is a known ocular pathogen, this may indicate the potential for these intracellular bacteria in *Acanthamoeba* to cause mixed and severe infections during AK. The presence of bacterial keratitis, or occurrence of bacteria as amoebal endosymbionts, could amplify *Acanthamoeba*’s virulence and promote polymicrobial keratitis. Identifying bacterial endosymbionts harboured by *Acanthamoeba* is important for improving accurate differential diagnostics and prognostic evaluations of *Acanthamoeba* keratitis.

## Supporting information

S1 Table Oligonucleotide probes used in this study for hybridization assay. (XLSX)

S2 Table Type III secretion system effector genes (exoU and exoS) in in endosymbiotic *P*. *aeruginosa* (AK1-PA). (XLSX)

S3 Table Click-&-Go plus 488 imaging reagents for alkDala labelling. (XLSX)

S4 Table *Acanthamoeba* keratitis (AK) infection monitoring sheet for day 1 to 6. (XLSX)

S5 Table Bruker Daltonik MALDI biotyper result. (XLSX)

S1 Fig Agarose gel images depicting PCR amplicons of *Acanthamoeba* isolates. (**a**) recovered from domestic tap water of an AK patient (AK1) and a corneal sample of another AK patient (AK11) and associated intracellular bacteria (**b**). Bands were visualised using 1% gel electrophoresis; primer set JDP1/2 (*Rns*) and 341Fw/785Rv (V3-4, 16S rRNA) yielded 450 bp and 444 bp amplicons, respectively. Positive controls: *A*. *castellanii* (ATCC 30868) and *E*. *coli* (ATCC 10798) for 18S rRNA and 16S rRNA PCR assays; and molecular grade water for negative.
(TIF)

S2 Fig Map showing locations of AK patient’s domestic tap water sample collection site (AK1-H_2_O) and hospital where corneal sample was collected (AK11). The map was created using ArcGIS version 10.7.1 (Esri, GIS, California, USA). The base layer of this map was retrieved from the Esri Basemap (www.arcgis.com/apps/mapviewer/index.html?webmap=ff52218580f94d89851563f50cd1a2b2), and boundary was drawn using Diva GIS (diva-gis.org/gdata). The author’s affiliated institute, UNSW Sydney, holds a valid license for ArcGIS software.
(TIF)

S3 Fig Neighbour-joining phylogenetic tree based on the DNA sequence of the nuclear small-subunit (18S) rRNA, the *Rns* gene of *Acanthamoeba* isolates. Two *Acanthamoeba* isolates (AK1—blue coloured and AK11-yellow coloured) of this study belonged to genotype T4F subclade. The reference genotype sequences were obtained from the NCBI database.
(TIF)

S4 Fig Unrooted neighbour-joining tree based on partial sequence of 16S rRNA (V3-4) analysis, showing the relationship of endosymbiotic *P*. *aeruginosa* (AK1-PA) isolate to closely related strains of *P*. *aeruginosa* (represented by PA in the figure). These strains were isolated from different sources (indicated by colour range in the figure) and highly matched in blast_n_ search.
(TIF)

S5 Fig A middle image of Z-stacks (>12 images) with the FITC channel (probe pB-383), showing *P*. *aeruginosa* cells inside *Acanthamoeba* trophozoites instead of sitting on the surface. White arrows indicate rod-shaped bacterial cells and red line (dotted) represents trophozoite plasma membrane border.
(TIF)

S6 Fig Agarose gel image showing PCR amplicon of *P*. *aeruginosa* (AK1-PA) positive for *exoU* gene (~2000 bp band size). The previous isolate *P*. *aeruginosa* 6206 (PA 6206) was used as a positive control for *exoU* PCR assay.
(TIF)

S7 Fig Propidium iodide staining of one week old human monocyte-derived macrophages (hMDMs) harvested from a healthy donor. The freshly harvested macrophage cells were used to examine the intracellular survival ability of *Acanthamoeba*-adapted *P*. *aeruginosa* (AK1-PA) and wild-type PA-01 strains. (TIF)

S8 Fig Boxplot showing the weight of rats (group A and B) measured during the experimental period (day 1 to 5). The boxplots display the smallest and largest values (the 25th and 75th quartiles), and the median. There was not significant change in weight of either group rats during the infection period (day 1 to 5). Statistical analyses were performed using unpaired *t*-test.
(TIF)

S9 Fig Agarose gel image showing PCR amplicons of cyst-like cells harvested from group B rats cornea which were stained with CFW and MUC5ac. The suspected cells were collected using non-contact laser pressure catapult (LPC) procedure using Laser Capture Microdissection (LCM), and whole gDNA was subsequently extracted. PCR was performed using *Acanthamoeba* genus specific primer pair (JDPFw/Rv). As positive controls, two *Acanthamoeba* isolates (Ac113 and MK05-H2O) were included in the PCR.
(TIF)

S10 Fig Histological observation (HE staining) was performed on Wistar rat corneas to study *Acanthamoeba* keratitis induced by intrastromal injection of amoebal trophozoites. (**A**) and *P*. *aeruginosa* (**B**). Corneas infected by *Acanthamoeba* (AK10) alone (group C) showed a few areas of RBCs accumulation with the epithelium and endothelium slightly disorganized (**A**). In group D, the stromal region infected by *P*. *aeruginosa* exhibited a few necrosis like structures, RBCs, desquamated cells, inflammatory infiltrate, and visible disorganization of corneal epithelium and stromal structure (**B**). Indicators: White arrow, corneal epithelium; Yellow arrow: RBCs; Asterisk (*): Necrosis like structures with mild inflammatory infiltrate. Scale bar represents 15μm.
(TIF)

S11 Fig Boxplot showing the weight of rats (group C and D) measured during the *Acanthamoeba* keratitis experimental period (day 1 to 5). The boxplots display the smallest and largest values (the 25th and 75th quartiles), and the median. There was not significant change in weight of either group rats during the infection period (day 1 to 5). Statistical analyses were performed using unpaired *t*-test.
(TIF)
