# Supplementary figures and images for: The role of naturally acquired intracellular Pseudomonas aeruginosa in the development of Acanthamoeba keratitis in an animal model
Source: PLoS Negl Trop Dis. 2024 Jan 2;18(1):e0011878. doi: 10.1371/journal.pntd.0011878 (PMC10795995; doi:10.1371/journal.pntd.0011878)

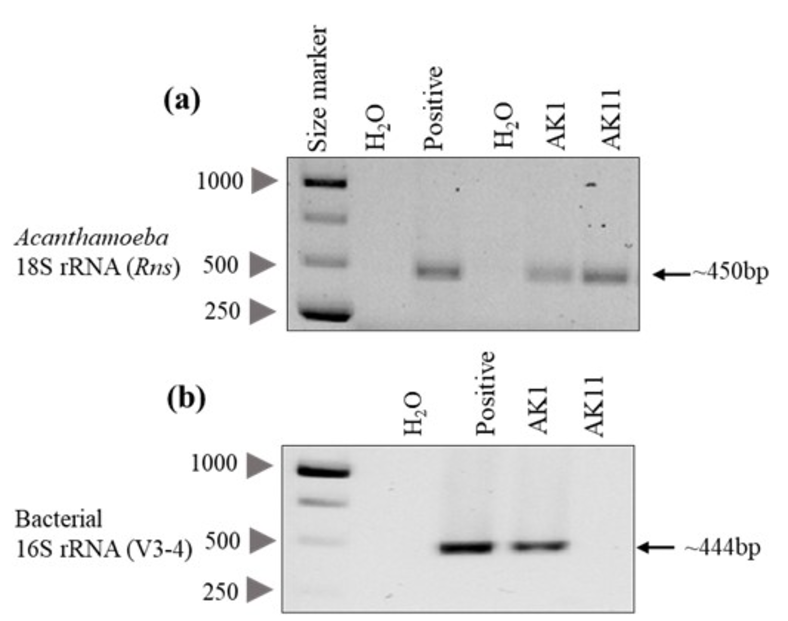

Supplement: S1 Fig — (a) recovered from domestic tap water of an AK patient (AK1) and a corneal sample of another AK patient (AK11) and associated intracellular bacteria (b). Bands were visualised using 1% gel electrophoresis; primer set JDP1/2 (Rns) and 341Fw/785Rv (V3-4, 16S rRNA) yielded 450 bp and 444 bp amplicons, respectively. Positive controls: A. castellanii (ATCC 30868) and E. coli (ATCC 10798) for 18S rRNA and 16S rRNA PCR assays; and molecular grade water for negative. (TIF) [file pntd.0011878.s006.tif]

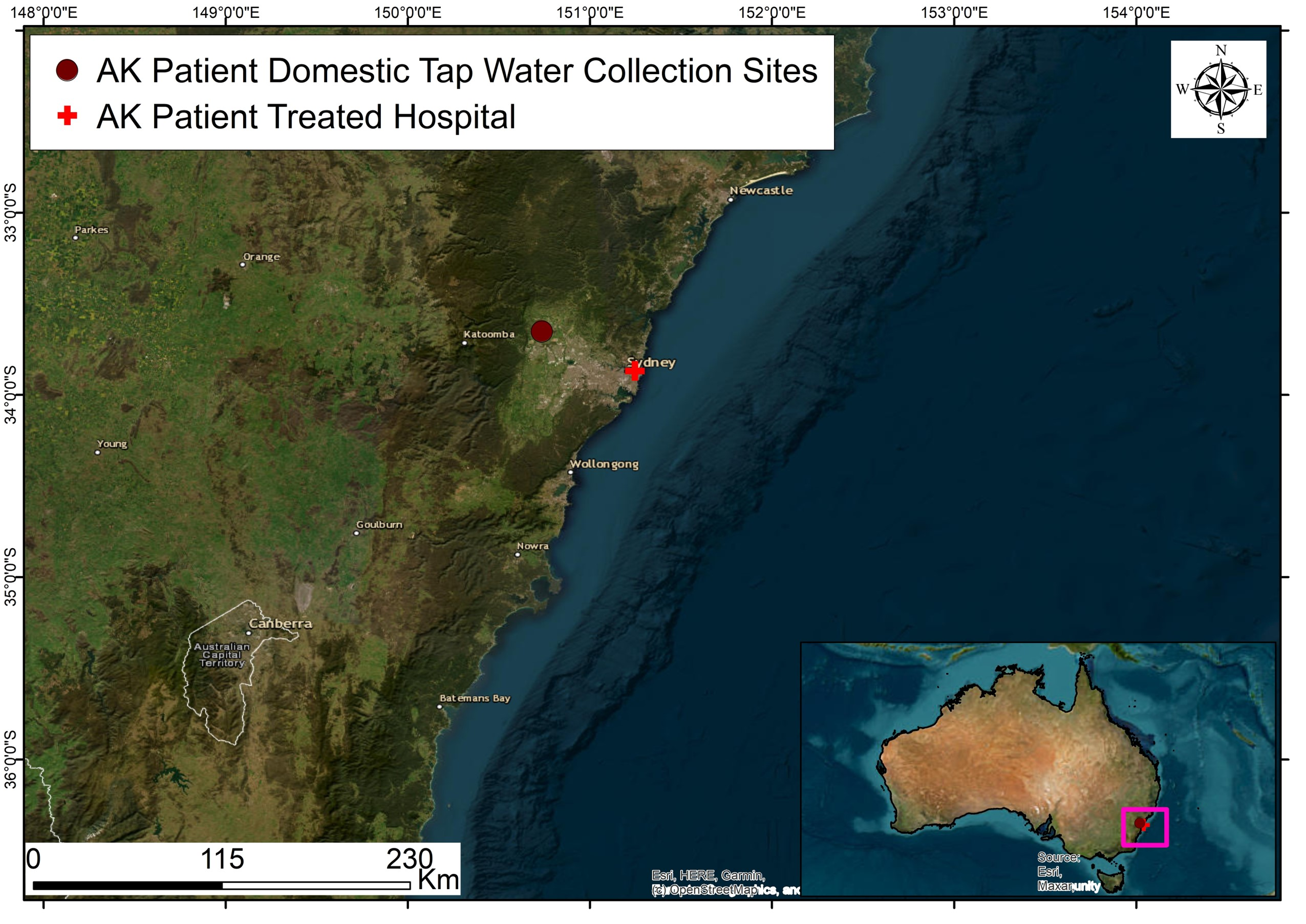

Supplement: S2 Fig — The base layer of this map was retrieved from the Esri Basemap (www.arcgis.com/apps/mapviewer/index.html?webmap=ff52218580f94d89851563f50cd1a2b2), and boundary was drawn using Diva GIS (diva-gis.org/gdata). The author’s affiliated institute, UNSW Sydney, holds a valid license for ArcGIS software. (TIF) [file pntd.0011878.s007.tif]

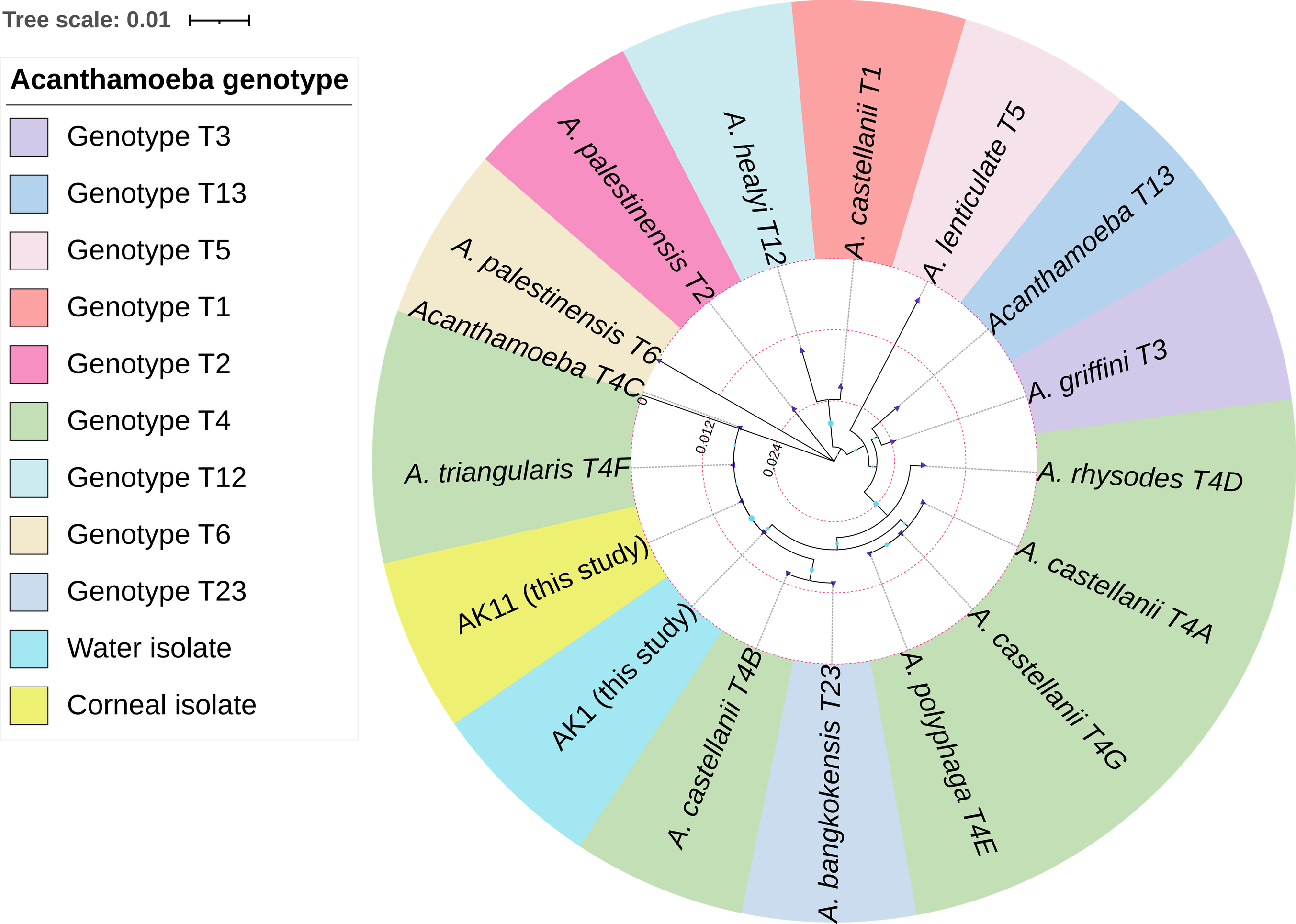

Supplement: S3 Fig — Two Acanthamoeba isolates (AK1—blue coloured and AK11-yellow coloured) of this study belonged to genotype T4F subclade. The reference genotype sequences were obtained from the NCBI database. (TIF) [file pntd.0011878.s008.tif]

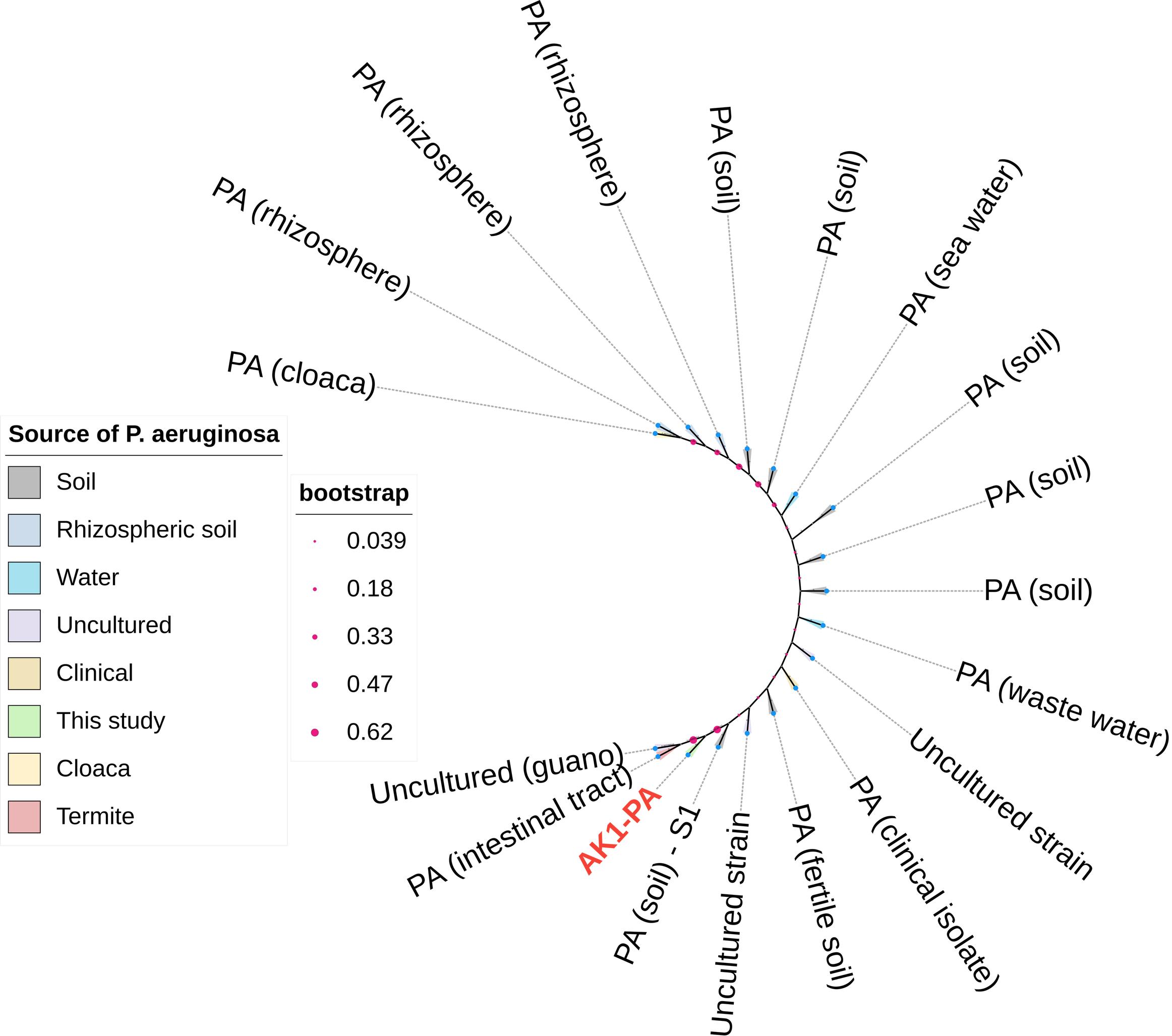

Supplement: S4 Fig — These strains were isolated from different sources (indicated by colour range in the figure) and highly matched in blastn search. (TIF) [file pntd.0011878.s009.tif]

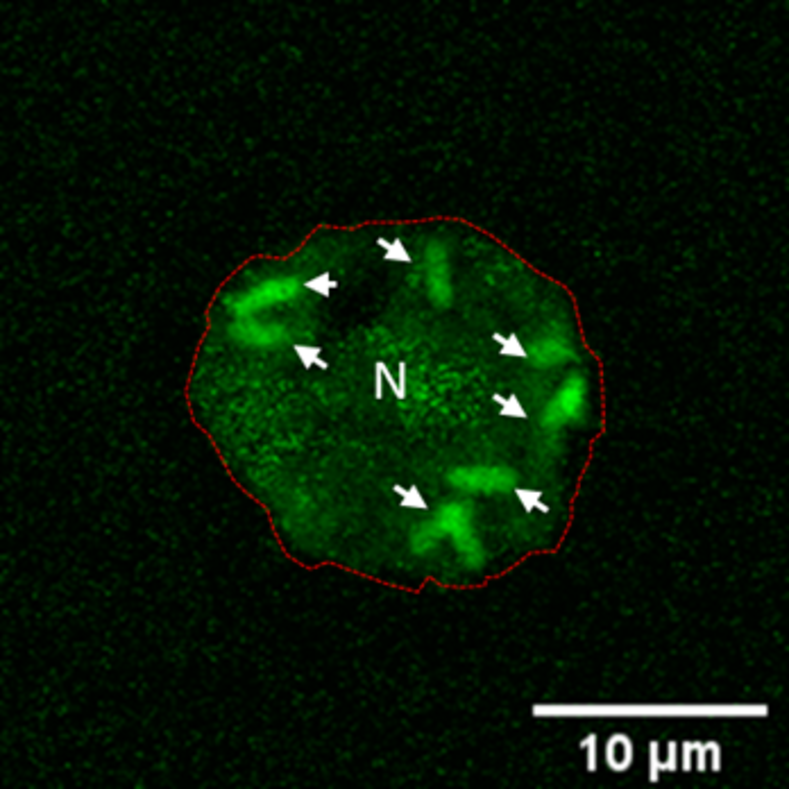

Supplement: S5 Fig — White arrows indicate rod-shaped bacterial cells and red line (dotted) represents trophozoite plasma membrane border. (TIF) [file pntd.0011878.s010.tif]

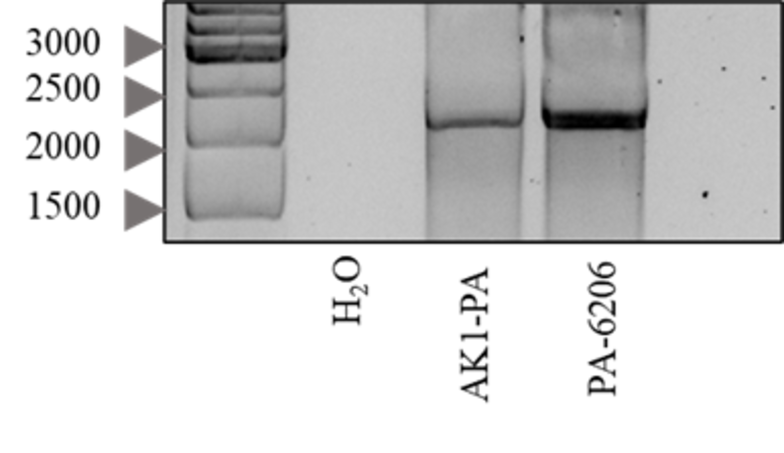

Supplement: S6 Fig — The previous isolate P. aeruginosa 6206 (PA 6206) was used as a positive control for exoU PCR assay. (TIF) [file pntd.0011878.s011.tif]

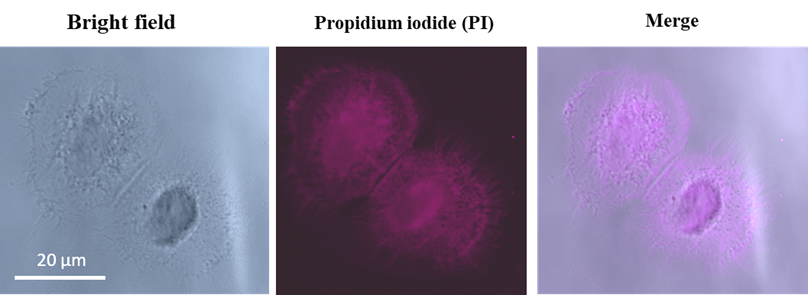

Supplement: S7 Fig — (TIF) [file pntd.0011878.s012.tif]

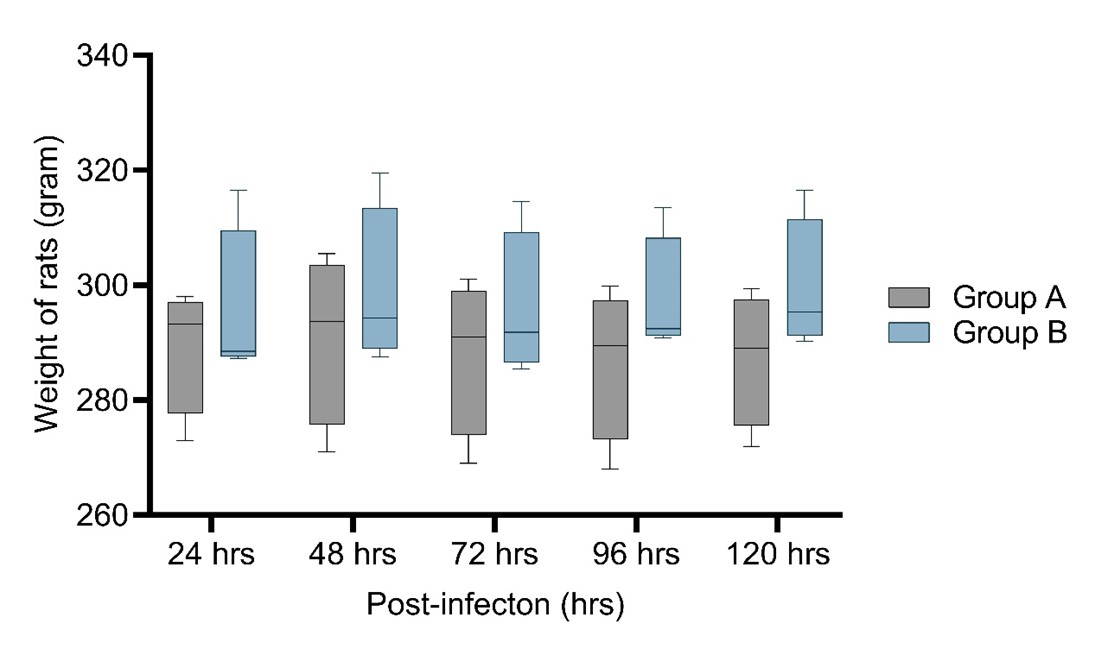

Supplement: S8 Fig — Boxplot showing the weight of rats (group A and B) measured during the experimental period (day 1 to 5). The boxplots display the smallest and largest values (the 25th and 75th quartiles), and the median. There was not significant change in weight of either group rats during the infection period (day 1 to 5). Statistical analyses were performed using unpaired t-test. (TIF) [file pntd.0011878.s013.tif]

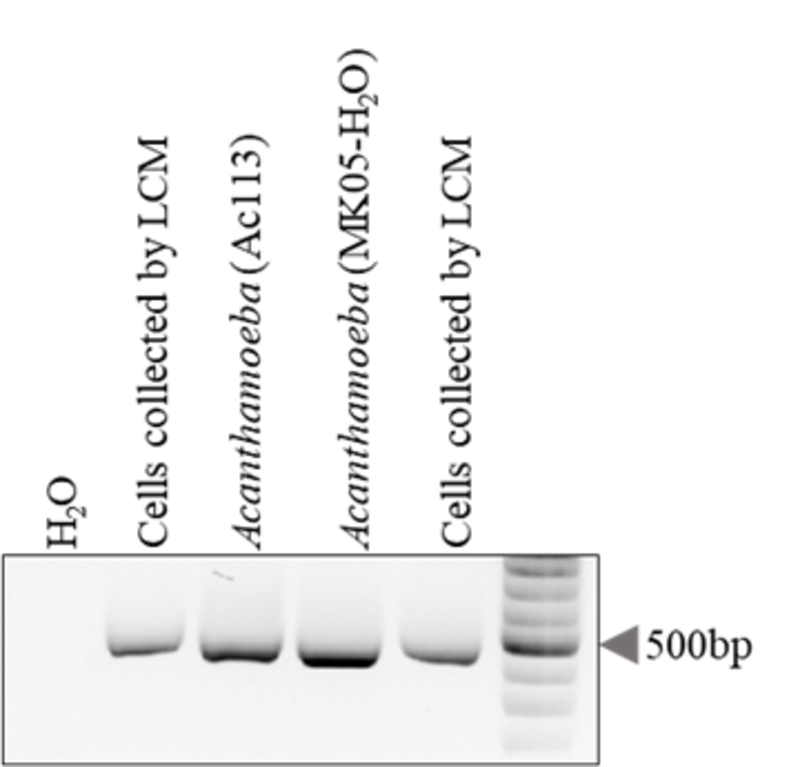

Supplement: S9 Fig — The suspected cells were collected using non-contact laser pressure catapult (LPC) procedure using Laser Capture Microdissection (LCM), and whole gDNA was subsequently extracted. PCR was performed using Acanthamoeba genus specific primer pair (JDPFw/Rv). As positive controls, two Acanthamoeba isolates (Ac113 and MK05-H2O) were included in the PCR. (TIF) [file pntd.0011878.s014.tif]

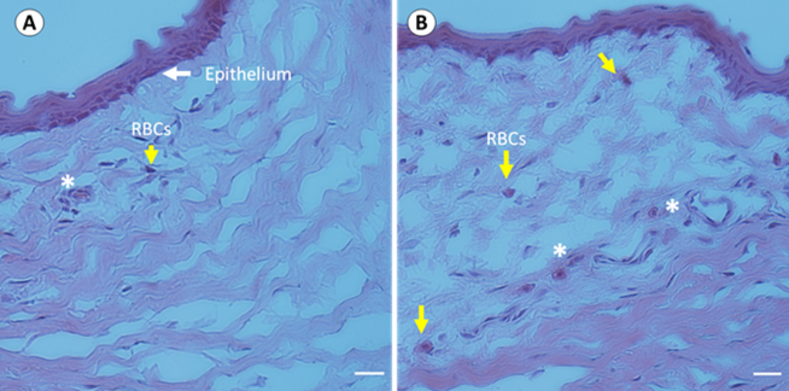

Supplement: S10 Fig — (A) and P. aeruginosa (B). Corneas infected by Acanthamoeba (AK10) alone (group C) showed a few areas of RBCs accumulation with the epithelium and endothelium slightly disorganized (A). In group D, the stromal region infected by P. aeruginosa exhibited a few necrosis like structures, RBCs, desquamated cells, inflammatory infiltrate, and visible disorganization of corneal epithelium and stromal structure (B). Indicators: White arrow, corneal epithelium; Yellow arrow: RBCs; Asterisk (*): Necrosis like structures with mild inflammatory infiltrate. Scale bar represents 15μm. (TIF) [file pntd.0011878.s015.tif]

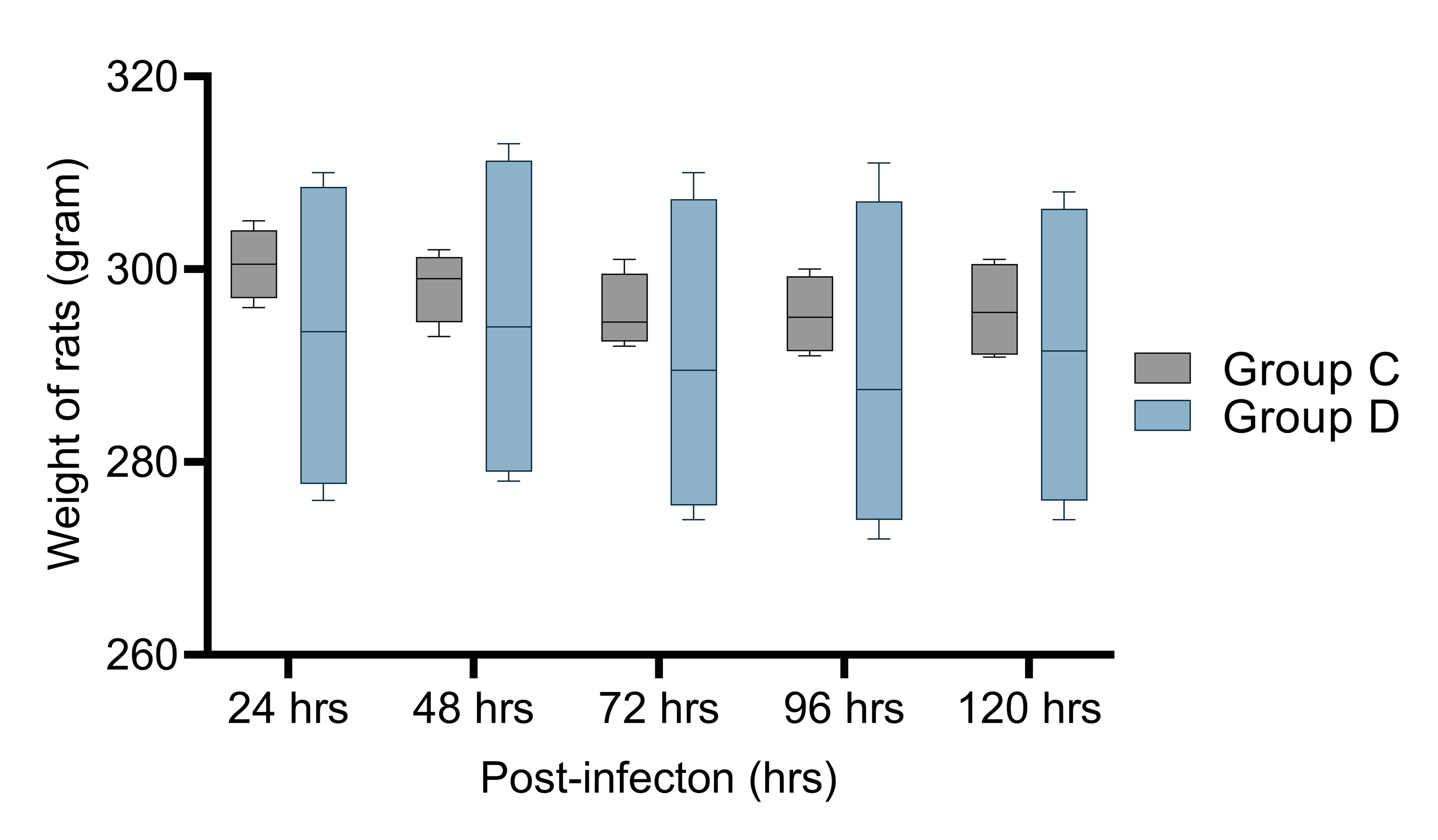

Supplement: S11 Fig — The boxplots display the smallest and largest values (the 25th and 75th quartiles), and the median. There was not significant change in weight of either group rats during the infection period (day 1 to 5). Statistical analyses were performed using unpaired t-test. (TIF) [file pntd.0011878.s016.tif]
